# Origin of a novel protein-coding gene family with similar signal sequence in *Schistosoma japonicum*

**DOI:** 10.1186/1471-2164-13-260

**Published:** 2012-06-20

**Authors:** Evaristus Chibunna Mbanefo, Yu Chuanxin, Mihoko Kikuchi, Mohammed Nasir Shuaibu, Daniel Boamah, Masashi Kirinoki, Naoko Hayashi, Yuichi Chigusa, Yoshio Osada, Shinjiro Hamano, Kenji Hirayama

**Affiliations:** 1 Department of Immunogenetics, Institute of Tropical Medicine (NEKKEN), and Global COE Program, Nagasaki University, 1-12-4 Sakamoto, 852-8523, Nagasaki, Japan; 2 Laboratory on Technology for Parasitic Disease Prevention and Control, Jiangsu Institute of Parasitic Diseases, 117 Yangxiang, Meiyuan, Wuxi, 214064, People's Republic of China; 3 Laboratory of Tropical Medicine and Parasitology, Dokkyo Medical University, Tochigi, Japan; 4 Department of Immunology and Parasitology, The University of Occupational and Environmental Health, Kitakyushu, Japan; 5 Department of Parasitology, Institute of Tropical Medicine (NEKKEN), and Global COE Program, Nagasaki University, 1-12-4 Sakamoto, 852-8523, Nagasaki, Japan; 6 Department of Parasitology and Entomology, Faculty of Bioscience, Nnamdi Azikiwe University, P.M.B. 5025, Awka, Nigeria

**Keywords:** Signal sequence trap, *Schistosoma japonicum*, Repetitive elements, Gene duplication, Secreted proteins, Non-allelic homologous recombination

## Abstract

**Background:**

Evolution of novel protein-coding genes is the bedrock of adaptive evolution. Recently, we identified six protein-coding genes with similar signal sequence from *Schistosoma japonicum* egg stage mRNA using signal sequence trap (SST). To find the mechanism underlying the origination of these genes with similar core promoter regions and signal sequence, we adopted an integrated approach utilizing whole genome, transcriptome and proteome database BLAST queries, other bioinformatics tools, and molecular analyses.

**Results:**

Our data, in combination with database analyses showed evidences of expression of these genes both at the mRNA and protein levels exclusively in all developmental stages of *S. japonicum*. The signal sequence motif was identified in 27 distinct *S. japonicum* UniGene entries with multiple mRNA transcripts, and in 34 genome contigs distributed within 18 scaffolds with evidence of genome-wide dispersion. No homolog of these genes or similar domain was found in deposited data from any other organism. We observed preponderance of flanking repetitive elements (REs), albeit partial copies, especially of the *RTE*-like and *Perere* class at either side of the duplication source locus. The role of REs as major mediators of DNA-level recombination leading to dispersive duplication is discussed with evidence from our analyses. We also identified a stepwise pathway towards functional selection in evolving genes by alternative splicing. Equally, the possible transcription models of some protein-coding representatives of the duplicons are presented with evidence of expression *in vitro*.

**Conclusion:**

Our findings contribute to the accumulating evidence of the role of REs in the generation of evolutionary novelties in organisms’ genomes.

## Background

Evolutionary novelties generated as an upshot of the “nascence” of new protein-coding genes are the bedrock of adaptive evolution and acquisition of novel molecular functions. The ever-growing vast and diverse protein repertoire in organisms can be ascribed to these events, and may explain the increasing heterogeneity among organisms of otherwise common ancestry [1-5]. Since the pioneering definitive treatise on gene duplication by Ohno about four decades ago [6], geneticists and evolutionary biologists have advanced this traditional notion; creating remarkable insights into the composite patterns and underlying mechanisms of genetic innovations. Some of these mechanisms are illustrated in a supplementary figure (Additional file 1). The advent of the genomics era has most importantly armed scientists with a valuable tool to enhance discovery of the rather intriguing mechanisms underlying the “birth” of new genes [5].

Apart from the canonical gene duplication model as proposed by Ohno [6]; extensive studies in various organisms have not only elucidated other models of gene duplication, including “dispersed” duplication in addition to the more definitive “tandem” duplication [7-13]; but has also revealed multiple mechanisms leading to the emergence of new functional genes. These include but not limited to: recombination by exon shuffling or exon “scrambling” [4,14-18]; retrotransposition by retrotransposons yielding intronless chimeric genes [18-25]; transduction of genomic segments by transposable elements by skipping the characteristic weak polyadenylation signal in retrotransposons leading to the mobilization of adjacent genomic sequence; or may involve a repetitive element (RE) mediated DNA level recombination (DLR) by a non-allelic homologous recombination (NAHR) mechanism, in which the REs provide the requisite homologous sequences for the recombination of genomic sequences in a non-allelic manner [7,20,26-30]. Horizontal gene transfer between organisms although infrequent, can give rise to new genes in the recipient organism [31-33]. *De novo* origination of protein coding genes from previously non-coding genomic sequences is a very important mechanism previously underrated, but accumulating data in many organism show that this event occur more often than previously thought [2,3,34-40]. Equally, a new gene can arise from the fusion of two genes [1,3,22] or fission of a “parent” gene [41]. These mechanisms seldom operate singly as they frequently overlap, collaborating in the creation of nascent genes as depicted in the famous origins of *Jingwei* and *Sphinx* in *Drosophila* species [14,19].

*Schistosoma japonicum* along with *S. mansoni* and *S. haematobium* are the principal schistosome species causing human schistosomiasis. Uncharacteristic of other human invading schistosomes, *S. japonicum* is also able to infect several non-human mammalian hosts. While *S. japonicum* and *S. mansoni* inhabit the periportal veins and cause an intestinal form of the disease, characterized by liver granulomatous fibrosis as a consequence of host immune response to the eggs lodged in the hepatic sinusoids [42,43]; *S. haematobium* causes urinary schistosomiasis at the vesical bladder plexus. Although *S. japonicum* produces similar lesions like *S. mansoni*, the fibrotic lesions and hepatosplenomegaly, the most severe outcome of schistosomiasis, is relatively more frequent and severe in *S. japonicum*[44]. Also, in contrast to *S. mansoni* and *S. haematobium,* acute disease due to *S. japonicum* is common in endemic foci and is associated with severe and persistent manifestations that may rapidly progress the host mediated immunopathogenesis, terminating in a network of fibrotic lesions [45]. Secreted proteins from the parasite ova embolized in the liver of the host are accessible to the host immune cells being located at the host-parasite interface and thus constantly exposed to the host liver tissues. Such interactions play critical role in the initiation and progression of granuloma and fibrosis formation by mediating inflammation [42-45]. Secreted protein candidates thus, possess great potentials for application in interventions aimed at preventing severe hepatic pathogenesis [46,47] among other applications.

Nascent genes confer extra functional capacities for the organisms to confront the challenges of the ever dynamic environment, and may equally, albeit rarely, inflict some functional constraints. In any case, recently evolved characteristics could best be attributed to either: protein family or domains expansion, gene loss events [48], or more likely, evolution of new genes. *S. japonicum* relatively exhibit a higher degree of parasitism and dependence on host derived molecules and signals as inferred from genomic and transcriptomic studies [49-51]; it is able to infect a wide range of hosts, and produces relatively more severe pathogenesis [45]. While these could be attributed to a number of other factors including: selective pressure of parasite-host interactions, the extensive gene loss and protein domain elimination or expansion events observed in its genome and transcriptome [49]; the evolution of novel functional protein coding genes before and after the divergence from other members of the genus *Schistosoma* could account for these extra characteristics.

Here, we report putative evolutionary novel gene family of Asian schistosomes, *S. japonicum* on the premise that no homologs of the genes were found in the genome of its evolutionary close relatives in the genus *Schistosoma,* or in any other organism with a complete sequenced genome*.* The genes first caught our attention as genes bearing similar or same signal sequence from our previous work that identified some secreted protein coding genes from the eggs of *S. japonicum* using a signal sequence trap (SST) [47]. Given the available tools prior to the publication of the *S. japonicum* genome sequence, we had attributed this observation to some alternative or trans-splicing models. The present analysis was inspired by the availability of the invaluable tool presented by the recently published partially assembled genome of this parasite [49]. We adopted an integrated approach utilizing extensive BLAST queries and other bioinformatics tools, transcription and expression analyses, southern hybridization of genomic DNA and evolutionary analyses. We describe evidence of “genome-wide” dispersed duplication of a protein coding gene locus, which may have arisen recently from previously non-coding genomic sequence. The role of repetitive elements as major mediators of the dispersive duplication is analyzed and discussed. Detailed evidence of the potential transcription models of some protein-coding representatives of the duplicons with similar signal sequence is presented and supported by our observations. Finally, based on the identification of non-coding mRNA transcripts as alternatively spliced variants of protein coding mRNAs, we propose that the new genes could be under significant functional selection.

## Results and discussion

### Sequence characteristics of a novel protein-coding gene family with similar signal sequences in *S. japonicum*

**Figure 1:**
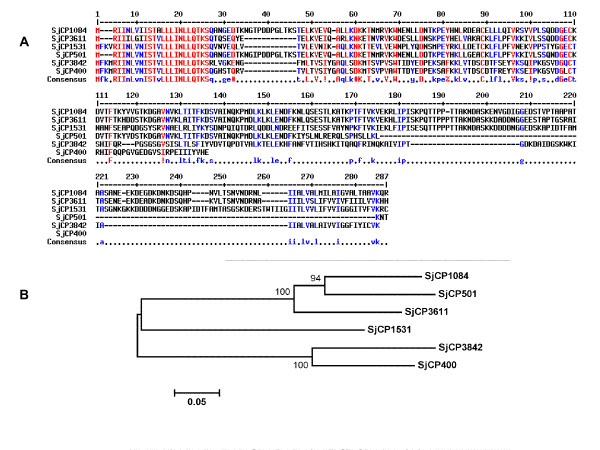
**Multiple alignments of protein sequences of the SST identified cDNAs showing similar signal peptide.** (**A**) The protein products of the original SST isolated *S. japonicum* egg cDNAs were aligned using ClustalW. The aligned sequences are limited to the candidates identified using SST, excluding database sequences. The N-terminal similar signal peptide is automatically colored red, indicating high similarity at the consensus sequence. Ostensibly, several other residues are also conserved and would be explored during the functional characterization. (**B**) The phylogenetic tree of the novel protein family identified using SST is shown here. The evolutionary history was inferred using the Minimum Evolution method. The evolutionary distances were computed using the p-distance method and are in the units of the number of amino acid differences per site. The analysis involved 6 amino acid sequences originally isolated using SST. Phylogenetic and evolutionary analyses were conducted on *MEGA5*[76].

To identify secreted proteins from the eggs of *S. japonicum*, we previously utilized a signal sequence trap (SST) and isolated at least 15 full length *S. japonicum* egg stage cDNAs encoding secreted or membrane binding proteins [47]. In addition, we observed that six of these genes have same or similar signal sequences (Table 1) from our analyses in [47]. Multiple alignment of the initial SST isolated messenger RNAs (mRNAs) is presented as a supplementary information (Additional file 2), while the multiple alignment of the corresponding protein sequences showing the similar signal peptides is presented in Figure 1 with the phylogenetic tree of the SST identified family members. Given the available tools at the time we made this observation, we had attributed this trend to some alternative splicing or trans-splicing models. Here, we took advantage of the recently characterized and published partial assembly of the genome sequence and transcriptome of *S. japonicum* to unravel the possible underlying mechanisms of signal sequence similarity among SST identified genes. BLASTN search on the whole non-redundant (nr) nucleotide collections and all expressed sequence tags (ESTs) in GenBank including the *S. japonicum* transcriptome using the similar signal sequence as query showed that a total of 181 mRNA sequences and 14 ESTs all belonging to *S. japonicum* bear the similar signal sequence. Based on information in the UniGene database that provides sets of transcript sequences that appear to come from the same transcription locus, these mRNA sequences with similar signal sequence were placed in 27 distinct UniGene entries (Table 2). By further sequence alignments of the returned mRNA sequences and information from UniGene, we grouped the mRNA transcripts according to their gene products and identified at least 7 distinct egg proteins, somula protein, 53 other hypothetical protein sequences and 10 non-coding mRNAs, all bearing the similar signal motif. All protein products of the mRNAs in the public database bearing the similar signal sequence were characteristically short, with one of them containing only 54 amino acids residues. A genome wide BLAST search using the similar signal sequence as query against all whole genome shotgun (WGS) reads also produced hits on 34 *S. japonicum* genome contigs (Table 3) distributed within 18 genome scaffolds (Table 4), thus confirming the existence of such sequences in the genome at multiple loci. These loci were non-redundant and non-overlapping as confirmed from the partially mapped scaffolds of this parasite’s genome accessible in GeneDB [52]. For clarity, we restricted further analyses to the initial cDNAs we had identified from our previous study using the SST.

**Table 1:** *SST isolated* S. japonicum*egg cDNAs with similar signal peptide

**Gene Products**	**GenBank cDNA Accession**	**GenBank Protein Accession**	S**ignal Peptide**
**SjCP1084**	AY570737 (1027 bp)	AAS68242 (271aa)	MRIINLVIISTALLLINLLQTKSQ
**SjCP3611**	AY570744 (983 bp)	AAS68249 (260aa)	MRIIILGIISTVLLLINLLQTKSQ
**SjCP501**	AY570753 (1038 bp)	AAS68258 (174aa)	MRIINLVNISTVLLLINLLQTKSQ
**SjCP3842**	AY570748 (854 bp)	AAS68253 (203aa)	MFKMRIINLVNISTVLLLINLLQTKSR
**SjCP400**	AY570756 (848 bp)	AAS68261 (124aa)	MFKMRIINLVNISTVLLLINLLQTKSQ
**SjCP1531**	AY570742 (1037 bp)	AAS68247 (274aa)	MFKVRIINLVNISTVLLLINLLQTKSQ

*SST: Signal Sequence Trap.

**Table 2:** *UniGene entries for* S. japonicum*mRNAs and ESTs bearing the similar signal sequence (n = 195)

**UniGene Name**	**UniGene ID (UID)**	**Set of likely mRNA transcripts (GenBank)**	**Gene Products (Database annotation)**
Sja.1526	1476162	AY814448, BU780442^est^	Egg protein SjCP3611
Sja.1611	1476247	FN317637, BU772954^est^	Hypothetical protein
Sja.1628	1476264	AY570742, FN320556, FN320555, FN320553, FN320552, FN320551, FN320550, FN320549	Egg protein SjCP1531
Sja.1676	1476312	AY570748^SST^, AY223245, AY222916, AY813542, EF127834, EF140742, FN323799, FN323800, FN323801, FN323803, FN323793, FN323792, FN323791, FN323790, FN323788, FN323785, FN323782, FN323781, FN323779, FN323778, FN323777, FN323776, FN323773, FN323772, FN323771, FN323770, FN323769, FN323768, FN323767, FN323766, FN323765, FN323764, FN323763, FN323762, BU772060^est^, BU766145^est^, CX862012^est^	Egg protein SjCP3842
Sja.2063	1476798	FN321064, FN321061	Egg protein SjCP1084
Sja.2065	1476800	AY570753^SST^, AY570744^SST^, AY814685, FN327232, FN327137, FN318042, FN321065, FN321060, FN321059, FN321058, FN321057, FN321056, FN321055, FN329815^nc^, BU768978^est^, BU780021^est^	Egg protein SjCP3611, Egg protein SjCP501, Hypothetical proteins
Sja.2070	1476805	AY599749^SST^	Egg protein SjCP1731
Sja.5326	2034920	FN326953, FN330298^nc^	Hypothetical protein
Sja.9771	2493712	AY570756^SST^, FN327121, FN327254, FN327253, FN327241, FN327233, FN327229, FN327224, FN327222, FN327216, FN327196, FN327185, FN327163, FN327158, FN327154, FN327129, FN327125, FN327115, FN327089, FN327083, FN327073, FN327057, FN327050, FN327049, FN327045, FN327042, FN327035, FN327022, FN327018, FN327014, FN327000, FN326998, FN326978, FN326973, FN326961, FN326960, FN326959, FN326930, FN326905, FN326883, FN326882, FN326881, FN326859, FN326857, FN326852, FN326851, FN326841, FN326831, FN326829, FN326822, FN326808, FN326801, FN326790, FN326770, FN326740, FN330540^nc^	Egg protein SjCP400, Somula protein
Sja.11083	2671933	AY915467, FN327219, FN327063, FN326828, FN326826, FN323794, FN323797, FN323798, FN323802, FN323789, FN323787, FN323786, FN323784, FN323783, FN323780, FN323774, FN323761, FN323760, FN323759, FN323758, FN323757, FN320521, FN320520, FN320519, FN320518, FN320517, FN320516, FN320515, FN320513	Egg protein SjCP3842, Hypothetical protein
Sja.11325	2672175	AY813755, FN320057, FN320056, FN320514, FN329566^nc^, BU768160^est^, BU774105^est^, BU770186^est^, BU779051^est^	Egg protein SjCP3842, Hypothetical protein
Sja.11840	2895838	FN327242, FN327131, FN327087, FN326854, BU776301^est^	Hypothetical protein
Sja.11891	2895889	AY813975, FN329814^nc^, BU769048^est^	Egg protein SjCP1084
Sja.13298	3987026	FN320059	Hypothetical protein
Sja.13324	3987052	AY570737^SST^, FN328299^nc^	Egg protein SjCP1084
Sja.13882	3987610	FN330716^nc^	None
Sja.13956	3987684	FN330422^nc^	None
Sja.14071	3987799	FN329677^nc^	None
Sja.14095	3987823	FN329269^nc^	None
Sja.14561	3988289	FN327139, FN323795, FN323796, FN323775	Egg protein SjCP3842
Sja.14562	3988290	FN327130, FN326955, FN326901	Egg protein SjCP1084
Sja.14565	3988293	FN327099	Egg protein SjCP1084
Sja.14614	3988342	FN320058	Hypothetical protein
Sja.14627	3988355	FN319007	Hypothetical protein
Sja.14941	3988669	FN320554	Hypothetical protein
Sja.15036	5233761	FN326786, FN318043, CX861530^est^	Hypothetical protein
Sja.15108	5233833	AY810465, FN321062	Hypothetical protein

* UniGene is a database of sets of transcript sequences that appear to come from the same transcription locus.

The original set of cDNAs we earlier identified using signal sequence trap bear the superscript tag (^**SST**^).

Transcripts with tags (^**est**^) and (^**nc**^) are expressed sequence tags (ESTs) and non-coding mRNAs respectively.

**Table 3:** ***Schistosoma japonicum*genome contigs containing the similar signal sequence (n = 34)**

*Contig [GenBank Accession No.]	**Transcription Strand**	**Size, kb**	**Signal Sequence coordinates**
**SJC_C002611 [CABF01002612]**	-	69.7	4848 – 4779
**SJC_C002621 [CABF01002622]**	-	0.6	276 – 205
**SJC_C002622 [CABF01002623]**	-	1.6	999 – 928
**SJC_C002627 [CABF01002628]**	-	3.0	2413 – 2342
**SJC_C002629 [CABF01002630]**	-	14.5	3856 – 3785
**SJC_C013669 [CABF01013761]**	-	6.8	3284 – 3217
**SJC_C019814 [CABF01020047]**	+	29.2	19023 – 19094
**SJC_C019817 [CABF01020050]**	-	10.1	6502 – 6431
**SJC_C019827 [CABF01020060]**	-	43.7	42511 – 42440
**SJC_C022876 [CABF01022876]**	-	12.4	9335 – 9264
**SJC_C022884 [CABF01022884]**	+	12.9	493 – 564
**SJC_C023364 [CABF01023364]**	-	12.4	10498 – 10427
**SJC_C025268 [CABF01025296]**	-	12.1	7860 – 7789
**SJC_C027826 [CABF01027854]**	+	4.3	433 – 504
**SJC_C027833 [CABF01027861]**	-	11.9	5768 – 5697
**SJC_C027838 [CABF01027866]**	+	19.0	12367 – 12438
**SJC_C032855 [CABF01032892]**	-	22.3	383 – 322
**SJC_C032859 [CABF01032896]**	-	9.6	4663 – 4602
**SJC_C043165 [CABF01043187]**	+	4.9	2484 – 2544
**SJC_C057153 [CABF01057161]**	+	2.8	337 – 408
**SJC_C061392 [CABF01061395]**	-	7.4	4411 – 4342
**SJC_C067189 [CABF01067176]**	+	4.9	388 – 459
**SJC_C067567 [CABF01067411]**	-	3.2	925 – 854
**SJC_C070280 [CABF01070230]**	-	6.8	271 – 200
**SJC_C072631 [CABF01072590]**	+	1.9	1646 – 1717
**SJC_C072632 [CABF01072591]**	+	2.6	446 – 517
**SJC_C073741 [CABF01073691]**	-	2.4	2389 – 2319
**SJC_C075160 [CABF01075030]**	-	6.7	5231 – 5160
**SJC_C076469 [CABF01076032]**	+	3.1	1295 – 1366
**SJC_C077101 [CABF01078976]**	-	1.1	656 – 585
**SJC_C080985 [CABF01080674]**	+	2.3	1094 – 1165
**SJC_C081391 [CABF01080757]**	-	2.1	1918 – 1847
**SJC_C081246 [CABF01080893]**	-	1.3	1131 – 1060
**SJC_C097686 [CABF01092393]**	+	5.6	4249 – 4320

(-) are contigs with ‘signal sequence’ on the negative strand (anti-sense) of the genome while

(+) are contigs with ‘signal sequence’ on the positive strand (sense) in the genome

*Contigs are representative of disperse duplicated gene loci. We indicated the ranges for the signal sequence motif.

**Table 4:** ***Schistosoma japonicum*Scaffolds containing the similar signal sequence (n = 18)**

**Scaffolds [GenBank Accession]**	**Contigs within the scaffolds**
**SJC_S000013 [FN330988]**	CABF01002611, CABF01002612, CABF01002622, CABF01002623, CABF01002628, CABF01002630
**SJC_S000219 [FN331192]**	CABF01020047, CABF01020050
**SJC_S000220 [FN331193]**	CABF01020060
**SJC_S000273 [FN331245]**	CABF01022876, CABF01022884
**SJC_S000284 [FN331256]**	CABF01023364
**SJC_S000329 [FN331301]**	CABF01025296
**SJC_S000394 [FN331366]**	CABF01027854, CABF01027861, CABF01027866
**SJC_S005820 [FN336777]**	CABF01067176
**SJC_S007785 [FN338731]**	CABF01070230
**SJC_S008639 [FN339578]**	CABF01072590, CABF01072591
**SJC_S009177 [FN340103]**	CABF01073691
**SJC_S010134 [FN341037]**	CABF01075030
**SJC_S011206 [FN342077]**	CABF01076032
**SJC_S011724 [FN342573]**	CABF01078976
**SJC_S014521 [FN345237]**	CABF01080674
**SJC_S014753 [FN345459]**	CABF01080893
**SJC_S014868 [FN345568]**	CABF01080757
**SJC_S026182 [FN354050]**	CABF01092393

To assess whether some homologs or at least some similar domains exist in other species, BLASTN and BLASTP searches using both the signal sequence and the entire coding sequences of the mRNAs and protein sequences as queries showed that these genes have no homologs in any other organism, but their expression in *S. japonicum* is supported by evidence from transcriptome and proteomic data. A search on several protein domain databases showed that although our candidates were classified in the same protein family with similar domains and assigned to a domain ID (ProDom:PD884968), no related domain or protein family was found in any other organism. The absence of these genes in the genome of *S. mansoni, S. haematobium* and other published genomes cannot possibly be attributed to sequencing gaps or annotation errors since the WGS sequencing approach is considerably reliable [49], and the fact that we adopted a multiple species approach covering the entire available sequenced genomes of all species makes this even more improbable [37,40]. Given the accumulating evidences of *de novo* origin of new genes from previously non-coding DNA sequences [2,34-40], we propose that the coding sequence of these genes may have recently originated *de novo* from previously non-coding DNA sequences in the ancestral forms, and subsequently duplicated and dispersed in the genome. This represents a more plausible interpretation than the improbable alternative hypothesis of concurrent gene deletion or inactivation in multiple ancestral lineages.

### Species and strain specific expression

**Figure 2:**
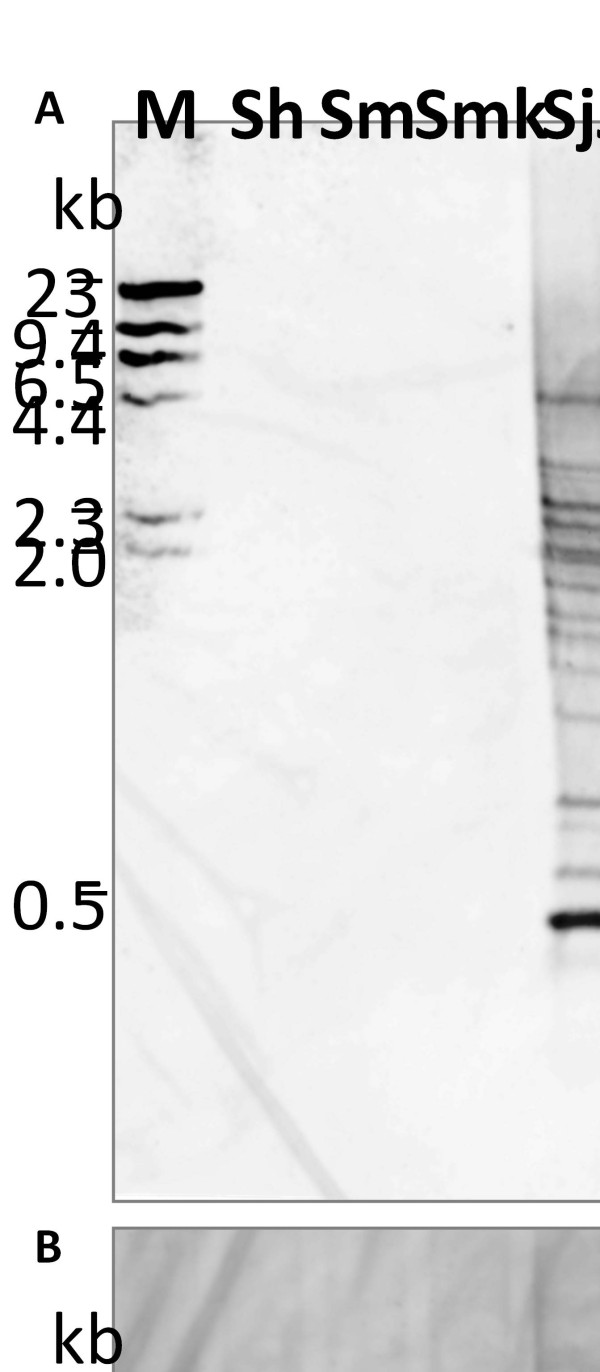
**Southern blotting confirms duplicated loci exclusively in*S. japonicum.*** (**A**) Southern hybridization with digoxigenin-labeled probes showing the presence of duplicated loci with several bands due to copies of the duplicated source locus. Lanes 2-9 corresponds to *Eco*RI + *Eco*RV double digested genomic DNA of different species and strains of Schistosoma (*S. haematobium*, *S. mansoni*, *S. mekongi*, *S. japonicum* (Japanese, Chinese, Philippines’ Leyte, Mindanao and Mindoro isolates). ‘M’ is Digoxigenin-labeled DNA molecular weight marker. Notice the differential banding pattern among different strains and between isolates of the same strain. (**B**) Same experiment as in (A) was replicated using a different pair of restriction enzymes (EcoRI + *Hind*III). Also inter-strain and intra-strain variation in the banding pattern is apparent.

To further exclude the possibility of false negative observations, we assessed the presence of the gene loci among different species and strains of *Schistosoma in vitro* using southern blots. This genomic locus and its duplicons was found to be exclusively present in all the strains of *S. japonicum* using southern hybridization experiment utilizing genomic DNA samples of different strains of *S. japonicum* (Japanese, Chinese and Philippines), and other species of *Schistosoma* including *S. mansoniS. haematobium* and *S. mekongi* (Figure 2), covering all the major clades in the genus. The result of southern hybridization using 462 base-pair digoxigenin labeled hybridization probe containing the similar signal sequence and designed to be specific to the gene loci under consideration showed that this genomic sequence was not found in any other species of *Schistosoma* except in all strains of *S. japonicum* analyzed. Several bands representing the duplicated loci are apparent in the hybridized blots (Figure 2). The analyzed samples is composed of representatives of the species complexes of this genus and further provide insight into the inter-species, intra-species and intra-strain variations that may exist among the members of the genus *Schistosoma*. In line with the widely accepted Asian origin hypothesis deduced from the evolutionary biogeography of this genus as inferred from evidences at the morphological, karyotype and molecular levels [53], it is highly plausible that this genomic sequence has recently evolved exclusively within the *S. japonicum* complex long after the divergence of the ancestors of the African species and other re-invading Asian species with origin from Africa [53,54]. The fact that this gene locus was completely lacking in other Asian species like *S. mekongi* of common ancestry with *S. japonicum* even throws more light on the most probable evolution of these other Asian species which are thought to have evolved from same ancestor or as descendants of *S. japonicum* based on mitochondrial gene arrangement [55]. Either *S. japonicum* and other Asian species in the *S. japonicum* group evolved independently from a common ancestor, or the evolution of this locus and the subsequent dispersed duplication occurred recently after the other Asian forms have diverged (see phylogenetic relationship in Additional file 3). An alternative explanation is that the gene was not fixed or was deleted from the genome of the other Asian and African species. Since the last hypothesis is highly unlikely, we concluded that our observation was a product of a newly evolved gene locus possibly from mutations or modifications on a previously non-coding sequence in the ancestral forms, which was subsequently severally amplified and dispersed in the genome of *S. japonicum* after all other species of the genus had diverged. Furthermore, a close look at the banding pattern of the restriction digested genomic DNA of different strains of *S. japonicum* as observed in the southern blotting result revealed that possible intra-species and intra-strain genetic variations could exist among the members of the species complex (Figure 2). Whether the *S. japonicum* complex (Japanese, Chinese, Philippines and animal infecting Formosa strains) is made up of four geographical strains, four subspecies or four independent biological species remains contentious. Be that as it may, this presents an interesting subject for further research and could be further explored using a wider array of isolates from different regions.

Nevertheless, while it is completely normal to verify this exclusive evolution and dispersed duplication hypothesis by confirming the physical localization of the gene loci in the genome and chromosomes by performing synteny analysis, we are unable to achieve this because we do not have access to a fully mapped chromosome information of the genome of *S. japonicum.* However, the distribution of the contigs and scaffolds bearing the similar signal sequence apparently suggests a dispersed distribution. To confirm this hypothesis and to exclude the possibility of overlapping among the loci, we generated the restriction map of six of the genome scaffolds bearing duplicated loci based on the information on the genome map, performed southern hybridization using restriction endonuclease digested genomic DNA from *S. japonicum* species and strains; and were able to match the expected probe binding fragment sizes with the observed bands on the hybridization blots (Additional file 4). Also an ancestral homolog is required for synteny analysis, however, we could not find a homolog in *S. mansoni*, another member of the genus with sequenced genome; and the genome of other more closely related species like *S. mekongi* and *S. malayensis* are not yet sequenced. Unless new evidences emerge from future updates in the sequenced genomes, we hold true that these genes have newly evolved, probably from modifications on previously non-coding ancestral DNA sequences and subsequently disperse duplicated. As opined in previous studies, the short length of our identified genes is an expected property of nascent genes because of improbability of evolution of long open reading frames (ORFs) and the complexity of intron splicing signal [38]. We expect these novel genes to be of functional significance since new genes tend to display accelerated sequence and structural changes towards neo-functionalization [1], and most newly characterized genes from other species have been shown to be characteristically functional [35,56]. Other workers showed that the common pathway for *de novo* protein-coding gene evolution involves a piece of DNA sequence to be transcribed via recruitment of all transcription core promoters, other elements and machines; followed by the acquisition of a translatable ORF through mutations or other sequence alteration mechanisms [2,35]. Together, our findings support the presence of these intrinsic features of novel genes in the identified candidates, including the gradual model of novel protein coding gene origination.

### Evidence of dispersed duplication from a source gene locus

**Figure 3:**
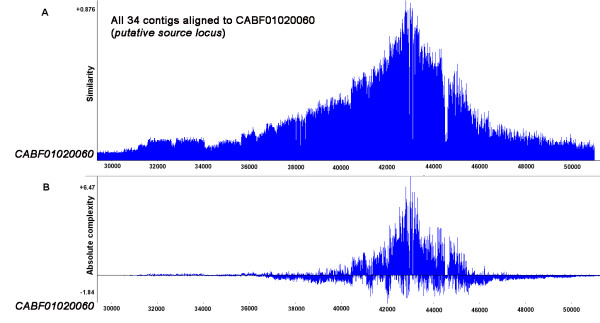
**Multi-alignments of 34** ***S. japonicum*genome contigs representing duplicated loci to assign putative source locus.** Graph shows the sequence similarity (**A**) and absolute complexity (**B**) of the DNA sequence of the 34 contigs in *S. japonicum* genome containing the duplicated loci. This multiple alignment was used to putatively assign the most prominent contig [GenBank:CABF01020060], the longest among the identified dataset (43.7 kb), which significantly covered the length of the other contigs as the putative duplication ‘source locus’. The curve shows the probable length of the duplicated locus, terminating with *RTE*-SJ at the 5` end (trimmed out in this figure) and *Perere* at the 3` end. ‘Similarity’ curve is a measure of the level of similarity of the aligned sequences. The y-axis on the ‘similarity’ curve will read ‘1’ when the sequences are 100 % similar in each position. The output shows the maximum score on the y-axis. Absolute complexity is a measure of the level of conservation or variability of nucleotides in the aligned sequences. It is a measure of the likelihood that the observed similarity did not occur by chance. The maximum positive score on the y-axis of the ‘absolute complexity’ curve is expected to be higher than the negative value to exclude any possibility that the observed similarity occurred by chance. The x-axes in both curves represent the nucleotide positions. The numbering of the nucleotides started at the 30000^th^ position in this figure because we trimmed output figure at the 5` end for ease of presentation.

The mechanisms behind dispersed duplication could be hidden within the DNA sequences of the duplicates or the adjacent flanking genomic sequences. In line with this, we explored the DNA sequences of the gene loci and the surrounding genomic sequences to identify possible mechanisms underlying dispersed duplication proposed in our hypothesis. A genome-wide BLAST search against WGS reads using the similar signal sequence as query returned 34 contigs of varying lengths and degrees of degradation (Table 3). By manually tracing these 34 contigs to the genome scaffolds, we found that they were distributed within 18 scaffolds (Table 4), apparently widely dispersed in the genome of *S. japonicum* as inferred from the genome map. To explore the mechanism of such dispersed duplication of a genomic sequence, a comparative analysis involving a parent gene in an ancestral species is often required. However, since we were unable to find any parental homolog in the available genome data and proteomes, and because gene duplication produces a diverse set of progeny loci with varied degrees of homology to an ancestral source locus when it exists [9], we performed a comparative sequence analysis on the 34 contigs as representatives of the gene loci. The result revealed a particular prominent contig in the *S. japonicum* WGS reads [GenBank: CABF01020060], the longest of the set of “duplicons” (43.7 kb), which significantly encompassed the length of the other contigs (Figure 3). CABF01020060 was therefore putatively selected as the duplication ‘source locus’ and utilized as such for most of the analyses performed in this study.

**Figure 4:**
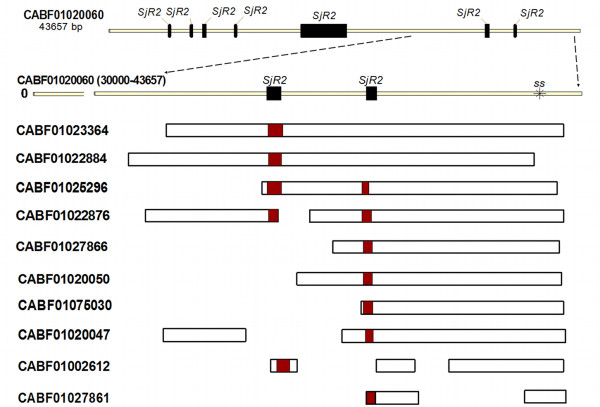
**Further evidence that the duplicated genes were duplicons of a single duplication source locus.** Apart from the prominent flanking copies of retrotransposons observed around the putative gene duplication source locus [GenBank:CABF01020060], other two short copies of the retrotransposon (*RTE_SJ*) are also found within introns in the coding region. We aligned the source locus with 10 of the duplicons and observed that both the signal sequence and these two partial copies of *RTE_SJ* are relatively aligned at same position, further indicating that the duplicated genes could have originated from a single source locus.

To investigate possible role by repetitive elements (REs) in mediating such dispersed duplication with a clue from previous studies [20,26-29], we performed repeat masking on the putative duplication source locus and the other 33 duplicons and observed a preponderance of flanking REs, especially of non-LTR class prominent of which were the *S. japonicum RTE (*retrotransposable element*)*-like retrotransposon (*SjR2*) and the *Perere* class of retrotransposons *(SjR1)* (Additional file 5). An almost full copy of *SjR2* was found upstream of the coding region of the putative source locus in addition to other six albeit partial copies of *SjR2*. Alignment of the other contigs to the putative duplication source locus revealed that both the dispersed similar signal sequence and the repeat elements are considerably aligned at very similar positions, further showing that they were likely duplicated from a single source locus. The fact that the duplicons are not absolutely homologous and the degenerative nature of the RE sequences suggests variation within members, typical of evolving genes (Figure 4). Because homology with the other duplicates did not terminate 3` of this putative source locus, we recruited and adjoined two contigs [GenBank:CABF01020061 and GenBank:CABF01020062] downstream of the putative source locus according to the genome assembly information, thereby creating flanking sequences of at least 5 kilo-basepairs on each side of the gene duplication source locus. This sequence was then aligned with the genome contigs and scaffolds to identify the exact point at which homology was lost, which could arguably represent the breakpoint of duplication. Further attempt to identify the exact breakpoints was not successful due to unfilled sequencing gaps in the scaffolds but examination of the downstream flanking sequence from the point where homology was terminated showed a prominent retrotransposon of the *Perere* class flanking the duplicated loci 3` of the locus (see short movie in Additional file 5). Taken together, our data show that the duplication source locus was flanked on either side by *RTE*-like and *Perere* class retrotransposons. These two classes of non-LTR retrotransposons have significantly high copy number, making up 12.63 % of the *S. japonicum* genome [49]. Different degrees of degeneracy of both the coding region and the flanking REs were observed in all the duplicated loci examined. This is consistent with the traditional view of the fate of new duplicons [6,9], which assumes a tendency to be lost because of genetic drift under natural evolution [29,57] while not precluding the possibility for some duplicates to evolve distinct functions either by sub-functionalization or neo-functionalization.

The role of repetitive elements (REs) in dispersed duplication of genomic sequences is fairly documented from previous studies in model organisms [15,20,27,28,30,58,59]. The precise mechanism of this retrotransposon mediated dispersed duplication is not clear but may likely involve RE-mediated DNA level recombination, most likely by non-allelic homologous recombination (NAHR), alternatively called ectopic recombination (see illustration in Additional file 6). Due to their extremely high copy numbers, REs create structural modifications in the genome by providing the requisite highly similar DNA sequences, initiating recombination between non-allelic elements [20,25,60], the result of which could be deletion, shuffling, duplication or transduction of a genomic DNA segment. Structural modifications introduced in the genome by NAHR mechanism can progress between non-homologous chromosomes (inter-chromosomal), between homologous chromosomes (inter-homologous or intra-chromosomal), between sister chromatids (inter-sister chromatid) or within a chromatid (intra-chromatid); giving rise to dispersed duplication of genomic segments, several forms of deletions or may create isodicentric chromosome by forming a mirrored segment in the chromosome by inversion. See detailed cartoon in Additional file 6. [60].

Many studies in other organisms have elucidated the role of REs in mediating sequence duplication, transduction and other structural variations by ectopic recombination mechanism. Notable among these is the human *Alu* element for which several reports suggest a role in mediating NAHR and other structural modifications in the human genome [7,20,61]. Yang *et al* found an excess of repetitive sequences proximate to the breakpoints of duplicated gene loci in the genome of the fruit fly *Drosophyla melanogaster,* and have suggested that a NAHR mechanism, mediated by REs accounted for the birth of the new duplicons [1,27]. Another study performed on human individuals concluded that NAHR accounted for over 40 % of detected genomic sequence duplications in the human genome [30]. Illegitimate recombination (IR), incomplete crossing over and non-homologous end joining (NHEJ) are other possible mechanisms of gene duplication by DNA-level recombination, but NAHR play a more significant role in producing typical dispersed duplications [1] while the other mechanisms in addition to NAHR are more likely to produce tandem duplicates. Although we could not clearly identify the exact breakpoints of the duplications at both ends still for lack of a reference ancestral homolog and partly due to sequencing gaps, the fact that homology among all the scaffolds examined uniformly terminated at the same point with *Perere* on the 3` end (Figure 3 and Additional file 5), and traces of the observed predominant retrotransposons (*SjR2)* was found at the exact positions as they occur in the putative source locus (Figure 4) confirm that these gene loci could be products of dispersed duplication from a single genomic source locus.

In addition to RE-mediated DNA-level recombination by NAHR, gene duplication events are also attributable to RE-mediated retrotransduction mechanism either on the 5` or 3` directions [27]. Xing *et al* and other groups have demonstrated the role of retrotransposons in the duplication of entire genes and creation of previously un-described genes by analyzing SVA (SINE, VNTR and Alu)-mediated retrotransduction events in the human genome [20,29]. However, we did not specifically identify any chimeric duplicon originating via a retrotransduction mechanism among our datasets. Furthermore, retrotransposons including *SjR2* characteristically encode reverse transcriptase and endonuclease, and can therefore transcribe and ‘paste’ a gene sequence into new locations in the genome [3,22,62]. However, retrotransposed genes are characteristically intronless since the introns are usually spliced out during the process of retrotransposition. Our duplicons retained their introns, although in some case some portion of the introns may have either degenerated or deleted during duplication and subsequent sequence modifications [3,22,63]. A further evidence that a retrotransposition mechanism is unlikely in our observed cases was that while retrotransposons would not duplicate the promoter regions of duplicated gene based on the process of transcription and insertion of retrocopies [1,57] which leaves the newly retrotransposed sequences to acquire new regulatory sequences from adjacent genes or through mutations in order to be functional [14,19,24]; the protein coding duplicons observed among our duplicated gene loci retained the same or similar core regulatory region and signal sequence as the source locus, suggesting that they may not have been products of retroposition and may equally explain the parallel assumption of coding potential at their new duplication loci without the need to form chimeric structures with adjacent genes.

### Evolution of translatable ORF and evidence of expression of duplicated genes

Some of the duplicons appear degenerative in homology and are relatively shorter than the source locus (Figure 3, also see Additional file 5) thus are consequently redundant and non-coding at the new locations as opined in the canonical view on the fate of new duplicons [6,9]; which assumes a tendency to be lost because of genetic drift under natural evolution [29,57]. However, our data provide evidence that some of the duplicons have evolved into protein coding genes with distinct products at their new loci, the fate of which could tend to either sub-functionalization to the source gene [8,64] or neo-functionalization by acquiring new distinct functions [9,65]. In addition to the two duplicons with alternative splicing variants, which we further explored in the next section, some representatives of the protein coding duplicons were depicted in a supplementary figure (Additional file 7). The nucleotide sequences of these genes are still appreciably similar but accumulation of mutations and other sequence modifications have given rise to novel protein coding ORFs, encoding putatively distinct products. We identified and mapped each cDNA sequence to the genomic contigs using information we generated from GeneMark and GeneQuest gene predictions [66] and confirmed by alignment of the cDNAs to the genomic sequences using NCBI *Spling* program. This approach was necessitated because the fully mapped and annotated genome of *S. japonicum* is not presently available in the public databases. Intriguingly, our results corroborate the available UniGene and GenBank entries. Nevertheless, it is notable that we only assessed the duplicated copies on the basis of possessing the similar signal sequence. There is possibility that some other duplicons from this source locus could be involved in initiating other forms of structural modifications at other loci when incorporated into the coding region of other genes, but this was not investigated here.

**Figure 5:**
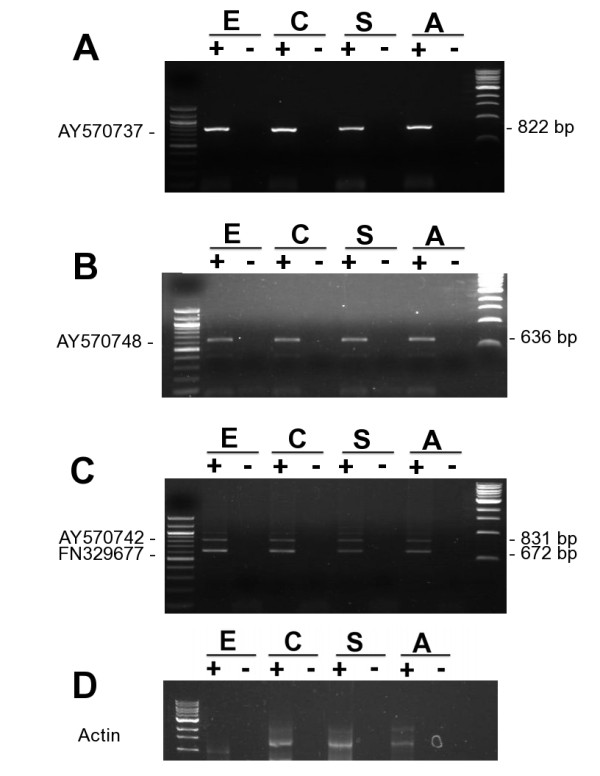
**RT-PCR showing expression patterns of some of the duplicons in the developmental stages of the parasite.** RT-PCR using cDNA libraries of the parasite egg (E), cercaria (C), schistosomula (S) and mixed sex adult (A) as template provide evidence of the transcription and expression of some of the duplicons. The pairs of primers used were designed to amplify the entire coding sequences of the mRNAs. No differential expression pattern was observed but quantitative expression levels were not investigated. (**A**) Evidence of expression of SjCP1084 protein coding mRNA [GenBank:AY570737], transcribed from the putative source locus [GenBank:CABF01020060]. See Table 2 for list of other similar transcripts. Notably, a non-coding transcript variant [GenBank:FN328299] can also be transcribed from the same locus. See Figure 6 and Additional file 8 for more details. (**B**) Expression of SjCP3842 protein coding mRNA [GenBank:AY570748] predictably transcribed from [GenBank:CABF01002612]. See Table 2 for list of other similar transcripts in the database. (**C**) Expression of SjCP1531 [GenBank:AY570742] predictably transcribed from [GenBank:CABF01023364]. See Table 2 for list of other similar transcripts. Notably, a non-coding transcript variant [GenBank:FN329677] can also be transcribed from the same locus (second band). See Figure 6 and Additional file 9 for details. (**D**) S. japonicum Actin gene was used as internal control to qualify the samples.

To provide evidence of the transcription and expression of the putative source gene locus and some of the duplicons, we performed developmental stage specific RT-PCR using primers that specifically amplify the coding regions of the candidate genes from the cDNA libraries of each stage of *S. japonicum*. RT-PCR results provide evidence of the transcription of some of the duplicons at their new genomic sites in addition to the source locus (Figure 5). The candidate genes analyzed did not show differential developmental stage specific expression, although we did not perform quantitative estimation of expression levels. It is possible that this group represents a potential new family of proteins with similar signal peptides in this zoonotic trematode, which possess other extra distinctive characteristics from other members of the genus *Schistosoma.* We are presently undertaking further research to fully characterize the identified novel protein-coding genes to provide insight into the functional and structural significance of this trend in the genome of *S. japonicum*. The protein products of some of these candidate genes have already been expressed in our laboratory and confirmed by the reactivity of the immune sera with the parasite crude antigen preparations. The data will be reported with the molecular and functional characterization information.

### Functional selection by alternative splicing

The precise recognition of exon-intron junction in a precursor mRNA (pre-mRNA) by the splicing machinery is central for the production of functional translatable mRNAs. However, there is often uncertainties in the choice of recognizable splice signals, resulting in a process termed alternative splicing [17], which enables the origination of multiple mRNA transcript variants from a single gene locus [67-69]. Alternative splicing mechanism could result in ‘intronization’ of an exon or ‘exonization’ of an intronic sequence. Ideally, the creation of an intron from a previously exonic sequence could lead to the loss of an ORF in coding genes. In evolving genes however, functional selection possibly by mutations may evolve the required splice signals and induce the intronization of an exon in a transcribed but non-coding mRNA gene sequence to create a translatable ORF encoding a functional protein. Conversely, while exonization of an intron could disrupt a translatable ORF in a coding gene, selective pressure may also evolve new splice signals within an intron to yield exons that could create a translatable ORF from a previously non-coding gene locus or a chimeric ORF from a protein-coding gene.

**Figure 6:**
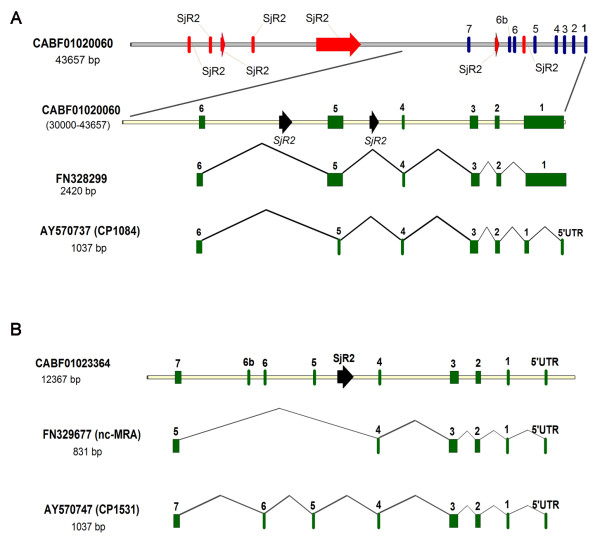
**Splice models of some duplicons with evidence of alternatively splicing.** (**A**) SjCP1084 protein coding mRNA [GenBank:AY570737] and a non-coding transcript [GenBank:FN328299] are products of alternative splicing. Based on gene prediction from the contigs using GeneQuest and GeneMark, and alignment of cDNAs to genome sequences using *Spling* program, we observed that two mRNA transcript variants were produced from [GenBank:CABF01020060]. An extra splice site was evolved in the first exon of the non-coding transcript [GenBank:FN328299]. When the splice site is recognized, an ORF encoding a protein coding mRNA [GenBank:AY570737] variant is created. The images were created from computer simulation of real DNA sequences using Vector NTI program. Also see a supplementary figure in Additional file 8 for more details. (**B**) SjCP1531 protein coding mRNA [GenBank:AY570742] and a non-coding transcript [GenBank:FN329677] are products of alternative splicing. Two mRNA transcript variants can be produced from a contig representing on of the duplicated loci [GenBank:CABF01023364]. Two extra splice sites were not utilized in the transcription of the non-coding transcript [GenBank:FN329677]. When the splice sites were recognized, exons 5 and 6 of a translatable ORF were created to produce a protein coding mRNA [GenBank:AY570742] variant. Refer to RT-PCR result in Figure 5 (C) where two bands of exact size and sequence as the two variants described above are apparent on the agarose gel electrophoresis image. Also see Additional file 9 for more details.

These two mechanisms have been shown from our observations to be capable of creating functional coding-genes from previously non-coding albeit transcribed mRNA sequences. We identified at least two classical evidences of alternative splicing and we propose that in addition to increasing coding potential and genomic diversity [68,69], alternative splicing can also be one of the driving forces of adaptive evolution; producing genetic novelties and functional selection. The most prominent example of alternative splicing was observed in the duplication source locus [GenBank:CABF01020060], which was found to be able to produce a protein-coding mRNA [GenBank:AY570737] in addition to a non-coding mRNA transcript variant [GenBank:FN328299] (Figure 6). An alignment of the DNA sequences of these two transcripts with details of this observation is presented in the on-line published supporting information (Additional file 8). UniGene entries also suggest that the two transcripts are from the same locus (Table 2). An extra intron donor and acceptor sites were found within the first exon of the non-coding mRNA transcript [GenBank:FN328299]. While the transcription model of the non-coding variant did not recognize the extra splice signals and thus retained the intron of about 1 kb, the coding mRNA variant [GenBank:AY570737] recognized the splice sites and created an ORF from the gene by splicing out an intron thereby giving rise to the 5`untranslated region (5`UTR) and the first exon of a protein-coding gene encoding a protein product of 271 residues (SjCP1084). Additionally, another pair of splice acceptor and donor sites evolving at exon 5 of the non-coding variant resulted in the splicing out of a portion of the exon, all contributing in creating a translatable ORF in the protein coding variant (See Additional file 8 for details).

On the other hand, exons 5 and 6 of a coding mRNA variant [GenBank:AY570742] predictably transcribed from one of the progeny loci [GenBank:CABF01023364] were skipped in a non-coding shorter variant [GenBank:FN329677] without a translatable ORF (Figure 6 and Additional file 9). We observed that the sequences of exons 5 and 6 were similar and was repeated five times *in tandem* within this locus, but only two copies of the tandemly duplicated potential exons were incorporated into the coding sequence of the mRNA to create exons 5 and 6 of a protein-coding ORF of 274 codons (SjCP1531). These results represent typical models of alternative splicing by intronization and exonization respectively.

Although in evolutionary perspective, intron retention that creates a translatable ORF is considered more plausible than the reverse process; our data show that both mechanisms are potentially possible. Other groups have also identified intron gains recently in mammalian and rodent retrogenes [68,69]. The identification of non-coding mRNA variant alternatively transcribed from a single gene locus with a protein coding mRNA (Figure 6) is evidence that a novel protein-coding gene can originate from previously transcribed regions that contain the necessary transcription elements and provide RNA material for a protein translation machine [2,39,68]. Exon repetition has also been observed from our data to exist in this organism and could be instrumental in expanding the organism`s coding potential. The ‘parallel’ expression of the non-coding variant alongside the protein-coding transcripts is of significance and could suggest further that the gene may have been recently evolved. Non-coding RNAs have also been shown to perform some regulatory roles at various levels during gene expression [2,68,70]. This could be further explored with our data set. In the two described cases in our analyses, we have treated the non-coding isoforms as evolutionally preceding the coding variants; nevertheless, the reverse could also be the case. In addition to these two cases, we also identified a two-nucleotide insertion into a non-coding mRNA sequence [GenBank: FN330540] that yielded the coding mRNA of schistosomula protein with the similar signal peptide, with many similar transcripts in the database. However, this last observation could be an artifact from sequencing error since the existence of the non-coding transcript was not traceable to the genomic sequence.

## Conclusions

We have passably delineated the possible mechanism that led to the identification of several protein coding genes with similar signal sequence, following lead from our work that isolated secreted proteins candidate genes using SST. A trend was described in the genome of *S. japonicum* whereby a ‘newly evolved’ gene served as a source locus for dispersed duplication events leading to the formation of several expressed genes with similar transcription core promoter region and signal sequence. We further found that the duplicated gene locus was flanked by non-long terminal repetitive elements (REs), especially of the *RTE-*like and *Perere* class. We therefore inferred that REs may have played an important role in this dispersed gene duplication by creating the requisite homologous DNA sequence that mediate a DNA-level recombination, most probably by a non-allelic homologous recombination (NAHR) mechanism. Our findings also provide evidence of logical sequential process of novel gene origination by evolution of transcription core elements followed by translatable ORF. While similar RE mediated phenomena had been observed in other organisms, unlike our dataset, most analyses have centered on the model organisms. Our data contribute to the accumulating evidence that REs mediate diverse recombination events leading to novel gene origination and other evolutionary novelties.

## Methods

### BLAST search

We had earlier identified a particular 81 nucleotides (27 amino acids) sequence, which was commonly utilized as signal sequence by several of our signal sequence trap (SST) isolated *S. japonicum* cDNAs (Table 1) [47]. The sequence of this signal sequence was employed as query to search for matches in the GenBank non-redundant nucleotide sequence database and expressed sequence tags (ESTs) database for all organisms using BLASTN program in National Center for Biotechnology Information (NCBI) Basic Local Alignment Search Tool (BLAST) [71]. A search on the NCBI UniGene database [72] that provides information on sets of transcript sequences that appear to come from the same transcription locus was performed to ascertain redundancy and group identified transcripts. For genome-wide searches, the same query sequence and program were used to search the WGS reads from all organisms with sequenced genomes deposited in the NCBI genome databases. In a similar search in the protein database, the amino acid and nucleotide sequence of the same signal sequence was used as query for BLASTP and BLASTX searches respectively. Conserved domain architecture searches on all translation products of the SST identified candidate genes were performed using the conserved domain architecture retrieval tool on NCBI website [73] and compared with same analyses on the ProDom database of protein domain families available online at [74].

### Multiple alignments

All multiple sequence alignments of DNA and protein sequences were performed in parallel with ClustalW on MegAlign program in Lasergene 7 DNASTAR software, NCBI bl2seq, COBALT multiple alignment programs, and *Multialin* interface software [75]. cDNA-to-genome sequence alignments were computed using the free NCBI *Spling* program [75]. The latest update of the *S. japonicum* genome map is accessible at [52]. Phylogenetic and molecular evolutionary analyses were conducted using *MEGA* version 5 [76].

### Gene prediction

Gene predictions were performed using the GeneQuest program (Lasergene 7 DNASTAR) to predict potential coding regions, starts, stops, acceptors and donor sites using Borodovsky matrix files for *Caenorhabditis elegans*; and the results compared with that of the Eukaryotic GeneMark.hmm [66] gene prediction server provided freely on the website of Georgia Institute of Technology, Atlanta, USA.

### Repeat masking

The whole sequences of all the genome contigs bearing the similar signal sequence were screened against a reference collection of repetitive DNA elements in the RepBase database available at the Genomic Information Research Institute website, using the CENSOR repeat masking software [77]. Sequence analysis figures were generated using real DNA sequences on Vector NTI Advanced 11.0 (Invitrogen).

### Designation of putative duplication source locus and probable breakpoint

Reference to a parent gene is required for accurate determination of duplication breakpoint. However, in absence of a reference homolog, we putatively selected the most prominent contig [GenBank:CABF01020060], the longest among the identified dataset (43.7 kb), which significantly covered the length of the other contigs (Figure 3, also see Additional file 5) as the putative duplication source locus and utilized it as such for most of the analyses performed in this study. When the contigs were aligned with the putative source locus, homology was not lost till the 3` end of the aligned sequences. We therefore recruited two contigs [GenBank:CABF01020061 and GenBank:CABF01020062] downstream of the source locus based on genome assembly information, thereby generating at least 5 kb flanking sequences on either side of the duplication source locus. This sequence was then aligned with the genome contigs and scaffolds to identify the exact point where sequence identity disappeared. This point was arguably chosen as the possible duplication breakpoint and utilized as such in our discussions. We further attempted to identify a recurrent consensus sequence at the breakpoints but this was hampered by several sequencing gaps in the partially assembled scaffolds.

### Parasites, genomic DNA and developmental stage mRNA samples

Chinese strain of *S. japonicum* (hereafter abbreviated as *Sj*) was obtained from Jiangsu Provincial Institute of Parasitic Diseases Wuxi, Jiangsu Province, PR China, while the Philippine and Japanese strains of *S. japonicum* in addition to *S. mekongi (Smk)* samples, were maintained in the Laboratory of Tropical Medicine and Parasitology, Dokkyo Medical University, Tochigi, Japan. *S. mansoni* (*Sm*) adult worms were maintained by, and kindly provided by the Department of Parasitology, Institute of Tropical Medicine, Nagasaki University, Japan. *S. haematobium* (*Sh*) sample was from Department of Immunology and Parasitology, University of Occupational and Environmental Health, Kitakyushu, Japan. Total genomic DNA was purified from cut tissues of mixed sex adult worms from different species of *Schistosoma* using QIAamp DNA Mini Kit (QIAGEN) according to the manufacturer’s instructions. Qualification and quantification of genomic DNA extract was assessed by gel electrophoresis and ND-1000 spectrophotometer (NanoDrop, USA). To obtain sufficient amount of genomic DNA for southern hybridization experiments, the whole genome of each sample was amplified using the GenomePhi DNA Amplification Kit (GE Healthcare) according to the manufacturer’s instructions. Equally, total RNA was extracted from parasite eggs, cercariae, 24 h cultured schistosomulae and adult worms of *S. japonicum* according to the instruction manual of PureLink Micro-to-midi total RNA Purification System Kit (Invitrogen).

### Reverse transcription polymerase chain reaction (RT-PCR)

mRNA from eggs, cercariae, 24 h culture schistosomulae and adult worms of the Chinese strain of *S. japonicum* was used for RT-PCR. The first strand cDNA was synthesized from the total RNA of each developmental stage by using oligo (dT) primer according to the instruction manual of High Capacity cDNA Reverse Transcription Kit (Applied Biosystems) and the resulting cDNA was used as template for RT-PCR. The *S. japonicum* actin gene was used for internal quality assurance. The cDNA sequences of some selected SST identified secreted candidate genes were amplified using pairs of sequence specific primers designed according to the *S. japonicum* transcriptome data [49] in the NCBI public database. All RT-PCR amplicons were analyzed using gel electrophoresis and confirmed by sequencing using the BigDye Terminator v1.1 Cycle Sequencing Kit (Applied Biosystem).

### Southern hybridization

Southern hybridization was performed following standard procedures [78] using the DIG nonradioactive labeling and detection system (Roche, Germany). Briefly, the hybridization probe labeled with DIG-dUTP was synthesized using PCR DIG synthesis kit (Roche, Germany) according to the manufacturer’s instructions, and labeling was confirmed by size disparity with unlabeled amplicon as a result of slower migration in agarose gel due to digoxigenin labeling. Genomic DNA from different species of *Schistosoma* (*Sh, Sm, Smk, Sj* Japanese (Yamanashi), *Sj* Chinese (Jiangsu) and *Sj* Philippines (Leyte, Mindanao and Mondoro isolates) were double digested with three different pairs of restriction enzymes (*Eco*RI + *Eco*RV, *Eco*RI + *Hind*III and *Bam*HI + *Hind*III) to achieve the best possible fragmentation of the genomic DNA. The digested genomic DNA fragments were electrophoresed through 1 % (w/v) agarose gel, depurinated in 250 mM HCl, and denatured by incubating in two changes of denaturing solution for 15 min each (0.5 M NaOH, 1.5 M NaCl). The gels were then neutralized by incubation in two changes of neutralizing solution (0.5 M Tris-HCl at pH7.5, 1.5 M NaCl) for 15 min each, and DNA was transferred to a positively charged nylon membrane (Roche, Germany) by capillary action overnight using 20x SSC solution (3 M NaCl, 300 mM sodium citrate at pH 7.0). The transferred DNA was fixed to the membrane by baking in an oven at 80 °C for 2 hours after rinsing briefly in 2x SSC. After prehybridizing the membrane in 10 ml hybridization buffer (5x SSC, 0.1 % N-lauroylsarcosine (w/v), 0.02 % SDS (w/v), 1 % blocking solution (Roche, Germany)) for 30mins in a hybridization bag, 5μl of the PCR generated hybridization probe was mixed in 50μl of double deionized water, denatured by boiling for 5mins and introduced into the hybridization bag and incubated overnight with shaking at 50 °C. The membrane was washed in two changes of low stringent wash buffer (2x SSC, 0.1 % SDS) for 5mins each at RT, and twice in high stringent wash buffers (0.5x SSC, 0.1 % SDS) for 15 min each at 65 °C. The hybridized probe was then detected using anti-Digoxigenin antibody (Roche, Germany) using CSPD as the chemiluminiscent substrate according to the manufacturer’s instructions. The blot was then visualized by exposing to chemiluminiscence for 10 min in a LAS-4000 mini image reader (Fujifilm).

## Abbreviations

SST, Signal sequence trap; NAHR, Non-allelic homologous recombination; DLR, DNA level recombination; NHEJ, Non-homologous end joining; IR, Illegitimate recombination; RE, Repetitive element; ORF, Open reading frame; WGS, Whole genome shotgun.

## Competing interests

The authors declare that they have no competing interests.

## Author contributions

ECM participated in the conception and design of the study, in-silico analyses, molecular experiments, data analysis and interpretation and drafted the manuscript. YC carried out the signal sequence trap (SST) and participated in in-silico analyses. MK participated in the design of the study, SST, in-silico analyses, molecular experiments and data interpretation. MNS participated in in-silico analyses, molecular experiments, data interpretation and revised the manuscript. DB participated in molecular experiments and data analyses. MK_2_, NH, YC_2_ and YO maintained parasite life cycle and participated in molecular experiments. SH participated in data interpretation, supervision and revised manuscript for intellectual content. KH participated in the conception and design of the study, SST, in-silico analyses, data interpretation, revised the manuscript and general coordination. All authors approved final version of the manuscript.

## Supplementary Material

Additional file 1 **Schematics of some of the mechanisms of novel gene origination.** Apart from the pioneering idea of gene duplication [6], there are other mechanisms by which new genes are born. These include but not limited to exon shuffling or exon “scrambling” (a) [4,14-18]; fission or fusion of genes (b) [1,3,22], horizontal gene transfer between organisms (c) [31-33], *de novo* origination of protein coding genes from previously non-coding sequences (d) [2,3,34-40], retrotransposition by retrotransposons yielding intronless chimeric genes (e) [18-25], transduction of adjacent DNA by transposable elements (f) or may involve a repetitive element mediated DNA level recombination by a non-allelic homologous recombination (NAHR) mechanism (g) [7,20,26-30]. The figure was adapted from [3]. Click here for file

Additional file 2 **Multiple alignments of signal sequence trap (SST) isolated cDNAs showing similar signal sequence.** The similar promoter region including the signal sequence is boxed. The two arrows indicate the ‘ATG’ start positions utilized in the transcript ORF of the candidate mRNA sequences. Click here for file

Additional file 3 **Phylogenetic tree of the genus*Schistosoma*showing the possible origination point of new duplicated genes.** The species phylogeny was adapted from [53] as inferred from DNA sequencing, comparative molecular genomics and karyotyping. This phylogenetic tree was manually simulated and thus the length of the branches does not estimate dates or time scale. The tree shows the *S. japonicum* clade and a representative each of the other clades in the genus including the species that reinvaded Asia from Africa. See review in [53]. Based on the result of the southern hybridization in Figure 2, the species and strains that contain the duplicated genes encoding products with similar signal sequence are colored green and we inferred that the most probable time point estimate (black dot) of the gene’s emergence could be after the other species in the *S. japonicum* group (in parenthesis) have diverged. Click here for file

Additional file 4 Expected fragments on restriction map of genome scaffolds correspond to bands on southern blots.
To confirm dispersed duplication hypothesis and to exclude the possibilty of overlapping among the loci, the restriction map of six of the genome scaffolds bearing duplicated loci were generated (A). Using same restriction endonuclease enzymes as in the generated maps, we performed southern hybridization using restriction digested genomic DNA from *S. japonicum* species and strains, and were able to match the expected fragment sizes with the observed bands on the hybridization blots. The contigs and the expected probe binding sites were labeled followed by their sequence ranges. We denoted the respective restriction digested fragments with probe binding site using alphabets with their expected restriction digestion product sizes in parenthesis (*E + E*: *Eco*RI + *Eco*RV; *E + H*: *Eco*RI + *Hind*III; *B + H* = *Bam*HI + *Hind*III). As shown in (B), we were able to match the expected fragment with the southern blot bands, labeled using their corresponding alphabetic codes. Probe binding site on the positive strand were colored ‘green’ while the antisense sites were colored ‘red’. The tiny vertical lines on the graphics represent the cutting sites of the selected restriction enzymes. The restriction map and the image were generated using DNADynamo sequence analysis software. Click here for file

Additional file 5 **Simulations using our raw data to show DNA-Level recombination mediated by REs by NAHR mechanism.** The movie created from a Powerpoint presentation (Additional file 10) represents the basic approach we utilized in our analysis to show evidence of DNA level recombination by a non-allelic homologous recombination mechanism. The raw data obtained from BLAST searches and RepBase repetitive element prediction report was used to present a simulation that demonstrates that the duplicated locus is flanked on 5` and 3` ends by retrotransposons of the classes *RTE_SJ* and *Perere* respectively. We proposed that these repetitive elements could have provided the requisite homologous stretch of DNA that is required for such DNA level recombination. NAHR can be inter-chromosomal, intra-chromosomal, inter-sister chromatid, or intra-chromatid to give rise to disperse duplicates of the intervening genomic locus. This movie was created from an original Powerpoint presentation (Additional file 10) Click here for file

Additional file 6 **A simplified illustration of repetitive element mediated DNA level non-allelic homologous recombination (NAHR).** Repetitive elements provide the requisite homologous DNA sequence for DNA level recombination between non-allelic pairs by a NAHR mechanism. NAHR can occur within a chromosome (intra-homologous chromosomal), between chromosomes (inter-chromosomal), between sister-chromatids or within a chromatid to give rise to disperse duplicates of the intervening genomic locus. The figure was adapted from [60]. Also see Additional file 1 for a cartoon of NAHR and other mechanisms of new gene origination, and [20,26-29,60] for review. Click here for file

Additional file 7 **Splicing models of some protein-coding representatives of the young duplicons.** Based on gene prediction from the contigs using GeneQuest and GeneMark and alignment of cDNAs to genome sequences using *Spling* program, we married the predicted products to the transcriptome database of this parasite and found that some of the duplicons are able to code for distinct gene products. Some of the transcription loci can encode two mRNA transcript variants. The significance of this was further explored in Figure 6. Click here for file

Additional file 8 **SjCP1084 protein coding mRNA [GenBank:AY570737] and a non-coding transcript [GenBank:FN328299] are products of alternative splicing.**Based on gene prediction from the contigs using GeneQuest and GeneMark, and alignment of cDNAs to genome sequences using *Spling*program, we observed that two mRNA transcript variants were produced from [GenBank:CABF01020060]. This figure is same as Figure 6 (A) but we have in addition presented the aligned sequence of the two transcripts showing details of alternative splicing. An extra splice site was evolved in the first exon of the non-coding transcript [GenBank:FN328299]. When the splice site is recognized, an ORF encoding SjCP1084 protein coding mRNA [GenBank:AY570737] variant is created. Click here for file

Additional file 9 **SjCP1531 protein coding mRNA [GenBank:AY570742] and a non-coding transcript [GenBank:FN329677] are products of alternative splicing.** Based on gene prediction from the contigs using GeneQuest and GeneMark, and alignment of cDNAs to genome sequences using *Spling* program, we observed that two mRNA transcript variants were produced from [GenBank:CABF01023364]. This figure is same as Figure 6 (B) but we have in addition provided the aligned sequence of the two transcripts showing details of alternative splicing. Two extra splice sites were not utilized in the transcription of the non-coding transcript [GenBank:FN329677]. When the splice sites were recognized, exons 5 and 6 of a translatable ORF were created to produce SjCP1531 protein coding mRNA [GenBank:AY570742] variant. Refer to RT-PCR result in Figure 5 (C) where two bands (exact size and sequence as the two variants described above) are seen on the agarose gel electrophoresis image. Click here for file

Additional file 10 **Simulations using our raw data to show DNA-Level recombination mediated by REs by NAHR mechanism.**This Powerpoint presentation was used to create the movie in (Additional file 5). Click here for file
