# Supplementary material for: Origin of a novel protein-coding gene family with similar signal sequence in Schistosoma japonicum
Source: BMC Genomics. 2012 Jun 20;13:260. doi: 10.1186/1471-2164-13-260 (PMC3434034; doi:10.1186/1471-2164-13-260)
Supplement: Additional file 1 — Schematics of some of the mechanisms of novel gene origination. Apart from the pioneering idea of gene duplication [6], there are other mechanisms by which new genes are born. These include but not limited to exon shuffling or exon “scrambling” (a) [4,14-18]; fission or fusion of genes (b) [1,3,22], horizontal gene transfer between organisms (c) [31-33], de novo origination of protein coding genes from previously non-coding sequences (d) [2,3,34-40], retrotransposition by retrotransposons yielding intronless chimeric genes (e) [18-25], transduction of adjacent DNA by transposable elements (f) or may involve a repetitive element mediated DNA level recombination by a non-allelic homologous recombination (NAHR) mechanism (g) [7,20,26-30]. The figure was adapted from [3]. [file 1471-2164-13-260-S1.pdf]

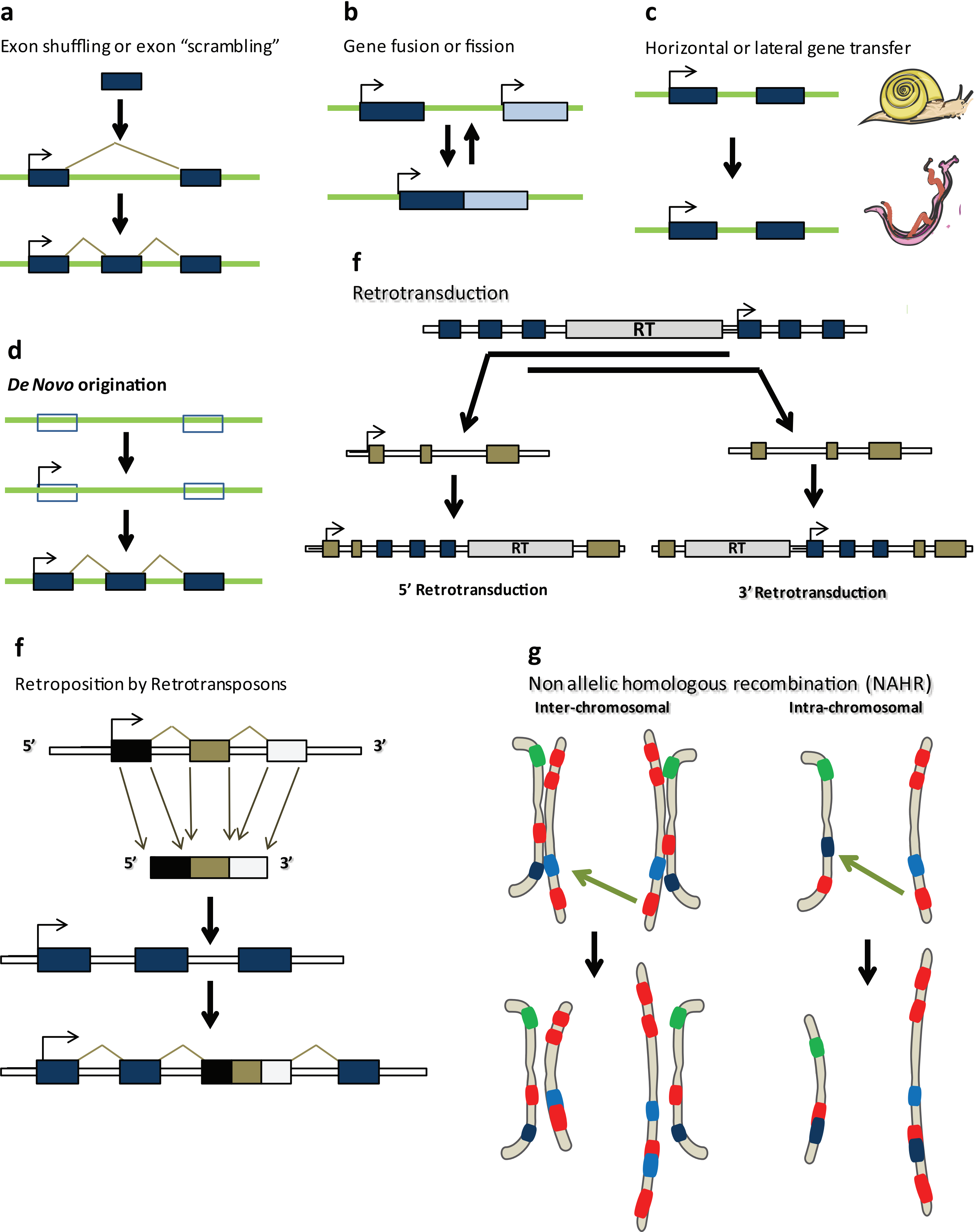

a
Exon shuffling or exon “scrambling”
b
Gene fusion or fission
c
Horizontal or lateral gene transfer
f
Retrotransduction
RT
RT
RT
5’ Retrotransduction
3’ Retrotransduction
d
De Novo origination
f
Retroposition by Retrotransposons
5’
3’
5’
3’
g
Non allelic homologous recombination (NAHR)
Inter-chromosomal
Intra-chromosomal
