# Supplementary material for: Origin of a novel protein-coding gene family with similar signal sequence in Schistosoma japonicum
Source: BMC Genomics. 2012 Jun 20;13:260. doi: 10.1186/1471-2164-13-260 (PMC3434034; doi:10.1186/1471-2164-13-260)
Supplement: Additional file 2 — Multiple alignments of signal sequence trap (SST) isolated cDNAs showing similar signal sequence. The similar promoter region including the signal sequence is boxed. The two arrows indicate the ‘ATG’ start positions utilized in the transcript ORF of the candidate mRNA sequences. [file 1471-2164-13-260-S2.pdf]

```
             1       10        20        30        40        50        60        70        80        90        100       110       120       130       140
             |--------+---------+---------+---------+---------+---------+---------+---------+---------+---------+---------+---------+---------+---------|
AY570737.1|                                           GAAAACTTAATCAGATTAAAAACAACTTTCAACTG--TGATCCGTCGAGATTCTTCTGTGAACGTTGTCGTATCTGACAAGCATTCAAAATGCGAATAAT
AY570753.1|  C----------------------ATAGGACTGAAGGAGTAGAAAACTTAATCAGATTAAAAACAACTTTCAGCTG--TGATCTGTCGGGATTCTTCTGTGAACGTTGTCGTATCTGACAAGCATTCAAAATGCGAATAAT
AY570744.1|                                           GAAAACTTAATCAGATTAAAAACAACTTTCAGCTG--TGATCCGTCGAGATTCTTCTGTGAACGTTTTCGTATCTGACAAGCATTCAAAATGCGAATAAT
AY570742.1|                                          GAAAAGCCTAATCAGATTACTATCATCCGTCAGCTG--TGATCCGTCGGGATTCTTCTGTGAACCTTTTTGTATCTGTTAAATGTTTAAAGTGCGAATTAT
AY570748.1|  CGACTGGAGCACGAGGACACTGACATGGACTGAAGGAGTAGAAAGCCTAATCAGATTACTATCAACCGTCAGCTG--TGATCAGTCAAGATTCTTCTGTGAACGTTTTCGTATCTGAGAAATGTTTAAAATGCGAATAAT
AY570756.1|  ACATAGGA-----------CTA---------GAAGGAGTAGAAAGCCTAATCAGATTACTATCAACCGTCAGCTAGTAGATCAGTCGAGATTTTTCTGTGAACGTTTTCGTATCTGAGAAATGTTTAAAATGCGAATAAT
 Consensus   ...............................gaaggagtagAAAgCcTAATCAGATTActAtCAaCcgTCAGCTg..tGATC.GTCgaGATTcTTCTGTGAACgTTtTcGTATCTGa.AAatgTTtAAAaTGCGAATaAT

             141     150       160       170       180       190       200       210       220       230       240       250       260       270       280
             |--------+---------+---------+---------+---------+---------+---------+---------+---------+---------+---------+---------+---------+---------|
AY570737.1|  CAACTTGGTGATTATTTCAACAGCGCTACTTCTAATTAACTTACTTCAAACGAAATCTCAAGCTAACGGTGAAGATACTAAAAATGGGACACCCGATGATCCTGGTTTAACAAAATCTACTGAGCTGAAGGTGGAAGTTC
AY570753.1|  CAATTTGGTAAATATTTCAACTGTGCTACTTCTGATTAACTTACTTCAAACGAAATCTCAAGCTAACGGTGAAGATACTAAAAATGGGATACCCGATGATCCTGGTTTAACAAAATCTACTGAGCTGAAGGTGGAAGTTC
AY570744.1|  CATCTTAGGGATTATTTCAACAGTGCTACTTTTGATTAACTTACTTCAAACAAAATCTCAAACTCAAAGTGAACAATATGAAA------------------------------------CTGAGCTGAAGGTGGAAATCC
AY570742.1|  CAACTTGGTGAATATTTCAACTGTGCTACTTCTGATTAACTTACTTCAAACGAAATCTCAAGTTAACGTTGAACAACTTGTAA------------------------------------CTGTCCTGGAGGTAAACATCA
AY570748.1|  CAACTTGGTGAATATTTCAACTGTGCTACTTCTGATAAACTTACTTCAAACGAAATCTCGACTTGTCGGTAAAGAAAATGGAT------------------------------------TCATGCTGACGGTGTCTATTT
AY570756.1|  CAACTTGGTGAATATTTCAACTGTGCTACTTCTGATTAACTTACTTCAAACGAAATCTCAAGGTCACAGTACACAACGTGTAA------------------------------------CTGTGCTGACGGTGTCTATTT
 Consensus   CAACTTGGTGAaTATTTCAACtGTGCTACTTCTGATtAACTTACTTCAAACGAAATCTCaAg.T.aCggTgaAcAa..Tg.Aa....................................ctgtgCTGaaGGTg.a.aTt.

             281     290       300       310       320       330       340       350       360       370       380       390       400       410       420
             |--------+---------+---------+---------+---------+---------+---------+---------+---------+---------+---------+---------+---------+---------|
AY570737.1|  AAG---CACTATTGAAGGATAAAAAGACAAACATGCGGGTCAAGTGGAATGAGAATTTGCTAGATAATACTAAGCCAGAATACCATAACCTTCGTGATGAAGCCTGTGAACTATTACTTCAAATTGTAAGGAGTGTAGTT
AY570753.1|  AAG---CACTATTGAAGGATAAAGAGACAAACATGCGGGTCAAGTGGAATGAGAATTTGCTAGATGATACTAAGCCAGAATACCATAAACTTCTTGGTGAAGCCTGTAAACTATTTCTTCCATTTGTAAAGAAAATCGTT
AY570744.1|  AAG---CACGATTGAAGAATAAAGAGACAAACGTTCGGGTCAAGTGGGATGAGAGCTTGCTAGATAATTCTAAGCCAGAATACCATGAACTTGTTGCTAAAGCCTGTAAACTATTTCTTCCATTTGTAAAGAAAATCGTT
AY570742.1|  AAG---CCCAATTAAAGAATAAAACGACAGAAGTTTTGGTCGAGTGGAATCCGCTTTACCAAGACAATTCTATGCCAGAATACCGTAAACTTCTTGATGAAACCTGTAAACTATTTCTTCCATTTGTAAACGAAAAAGTT
AY570748.1|  ATGGCGCACAGTTGTCCGATAAAATGACAAGCGTTCCGGTCTCCTGGACTATTGATTACGAAGATCCAGAAAAGTCAGCCTTCAAGAAACTTGTTACTGATTCCTGTGATACATTTAGTGAATACGTAAAATCTCAGATT
AY570756.1|  ATGGCGCACAGTTGTCCGATAAAATGACAAGCGTTCCGGTCGCCTGGACTACTGATTACGAAGATCCAGAAAAGTCAGCCTTCAAGAAACTTGTTACTGATTCCTGTGATACATTTCGTGAATACGTAAAATCTGAGATT
 Consensus   AaG...CaCaaTTgaaggATAAAa.GACAaacgTtc.GGTC.agTGGAaT..g.aTTaccaAGAt.at.ctAaGcCAGaaTaCcatAAACTTgTTgcTGAa.CCTGTgAactATTTctTcaATttGTAAA...t.a.gTT

             421     430       440       450       460       470       480       490       500       510       520       530       540       550       560
             |--------+---------+---------+---------+---------+---------+---------+---------+---------+---------+---------+---------+---------+---------|
AY570737.1|  CCTTTGAGTCAAGATGATGGAGAATGCAAAGATGTTACCTTTACAAAATATGTTGTTGGTACAAAAGATGGAGCTGTTAATGTGAAGT-TAACAATAACATTTAAAGACTCGGTTGCAATAAACCAAAAGCCTATGGATT
AY570753.1|  CTTTCGAGTCAAAATGATGGAGAATGCAAAGATGTTACCTTTACAAAATATGTTGATAGTACAAAAGATGGAGCTGTTAATGTGAAGT-TAACAATAACATTTAAAGACTCGGTTGCAATAAACCAAAAGCCTATGGATT
AY570744.1|  CTTTCGAGTCAAGATGATGGAGAATGCAAAGATGTTACCTTTACAAAACATGATGATAGTACAAAAGATGGCGCTGTTAATGTGAAGT-TAGCAATAACATTTAAAGACTCGGTTGCAATAAACCAAAAGCCTATGGATT
AY570742.1|  CCTCCGAGTACATATGGTGGTGAATGCACAAATGCTAACTTTAGTGAAGCTCCACAAGATGGATCATATTCTCGTGTTAATGCGGAGT-TACGTTTAATATATAAATACTCCGATAATCCACAAATTCAGACTGACAGGT
AY570748.1|  CCTAAAGGGTCAGTTGATGGACAATGCACAAGTCATATTTTTCAACGACCT------GGTTCAGGAAG---TGGTGTTAGTATAAG-CCTAACATTATCATTTATATACGTGGATGTAACACAACCTGACACTGTAGCGT
AY570756.1|  CCTAAAGGGTCAGTTGATGGACTATGCACACGTCATATTTTTCAACAACCT------GGTGTAGGAGAAGATGGTGTTAGTATAAGGCCTGAAATTATCATTTATGTACATGAATGAAATAAAACCTGACTATGCAGAGT
 Consensus   CcT..gaGt.cAgaTGaTGGagaATGCAcA.aTg.TA.cTTTa.a.aAccT......ggT.cA..Agatg.tggTGTTAaTgtgaagt.TaacatTAacATtTAaatACtcggaTg.aatAaAac.t.Ag.cTgt.gagT

             561     570       580       590       600       610       620       630       640       650       660       670       680       690       700
             |--------+---------+---------+---------+---------+---------+---------+---------+---------+---------+---------+---------+---------+---------|
AY570737.1|  TGAAATTAAAACTAGAAAATGATTTTAAAAATTTACAGTCTGAATCTACTTTGAAAGCAACTAAGCCCACATTCACTGTTAAAGTTGAAAAACGCTTGATTCCAATAAGCAAACCACAAACTACACCACCA---ACGACT
AY570753.1|  TGAAATTAAAACTAGAAAATGATTTTAAAA-TTTACAGTCTGAATCTACGTGAAAGGCAACTAAGCCCACATTCACTGTTAAAGTTGAAAAACACTTGATTCCAATAAGCAAACCACAAACTACACCACCACCAACGACT
AY570744.1|  TGAAATTAAAACTAGAAAATGATTTTAAAAATTTACAGTCTGAATCTACTTTGAAAGCAACTAAGCCCACATTCACTGTTAAAGTTGAAAAACACTTGATTCCAATAAGCAAACCACAAACTACACCACCACCAACGACT
AY570742.1|  TGCAGGATGACTTGAACGATCGAGAGGAATTCATCACATCCGAGTCTTCTTTCGTAGCATATAATCCCAAATTCACTGTTAAAATAGAAAAACTCTTCATTCCAATAAGCGAATCACAAACTACACCACCACCAACGACT
AY570748.1|  TAAAGACTGAATTAGAGAAACATTTTGCAAATTTCGTGACTATTCATTCTCATAAAATAACTCAAGCCCAATTCCGTATAAACCAAAAAGCAATAGTGATTCCAA-----------------------------------
AY570756.1|  TACTTAGTTACTTACGCGACAAATTCGCACATTTCCAGTTTACTTCTTCCCATAAAATAACTGAAGCCAGTTTCGGGGTATGCGAAGCAGATGTAGAGATTCCAA-----------------------------------
 Consensus   Tgaa...t.AatTaga.aAt.attttgaAaattTccagtctga.tcTtCtt..aaAgcAacTaA.cCCa.aTTCactgTtaaagtagaAaaactcttgATTCCAAtaagc.aa.cacaaactacaccacca...acgact

             701     710       720       730       740       750       760       770       780       790       800       810       820       830       840
             |--------+---------+---------+---------+---------+---------+---------+---------+---------+---------+---------+---------+---------+---------|
AY570737.1|  GCCAAGAATGACGCAAGTAAAGAAAACGTTGGTGACATTGGCGGTGAAGACTCAACAGTACCCACTGCTGCTCCTGCGACGGCAGCCTCCGCAAATGAAG---AAAAAGACGAAGGAGATAAAGATAACAAAGACTCACA
AY570753.1|  GCCAAGAATGACGCAAGTAAAAAAGACGCTGATGACAATGGCGGTGAAGAGTCAAAAGCACCCATTGGTACTTCTGCAATGACAGCCTCCGGAAGTAAAA---AAAAAGACGAAGCAGACAAAGATAACAAAGATTCCCA
AY570744.1|  GCCAAGAATGACGCAAGTAAAAAAGACGCTGATGACAATGGCGGTGAAGAGTCGACGGCACCCACTGGTTCCCGTGCGATAACAGCCTCCGAAAATGAAG---AAAAAGACGAAGCAGACAAAGATAACAAAGATTCCCA
AY570742.1|  GCCAAGAATGACGCAAGTAAAAAAGACGATGATGACAATGGCGGTGAAGACTCAAAAGCACCCATTGATACTTTTGCAATGACAGCCTCCGGAAATAAAGGTAAAAAAGACGATGATGACAATGGCGGTGAAGACTCAAA
AY570748.1|  -------------------CAGGAGACAAAGATGCAATTGACGG------------------------------------------------------------------------------------------------
AY570756.1|  -------------------GTGAAGACAAAGATGCAATTGACGG------------------------------------------------------------------------------------------------
 Consensus   gccaagaatgacgcaagtaaagaAGACgatGATGacAtTGgCGGtgaaga.tc.a..g.accca.tg.t.c...tgc.a...cagcctccg.aa.t.aa....aaaaagacga.g..ga.aa.g......aaga.tc..a

             841     850       860       870       880       890       900       910       920       930       940       950       960       970       980
             |--------+---------+---------+---------+---------+---------+---------+---------+---------+---------+---------+---------+---------+---------|
AY570737.1|  ACATCCCAATGTT-------------CTGACTTCAAACGTAAAC----------------GACAGAAAT-CTAATTATTGCATTGGTTGCACTGATGATCCTCGCGATTGGAGTTGCTTTAACGGCAGCCGTCAAACAAC
AY570753.1|  ACATCCCAAAGTT-------------CTGACTTCAAACGTAAAC----------------GACAGAAAT-GCAATTATTATATTGGTTTCACTGATCTTCGTCGTGATTGTATTCATTATAATTTTAGTCGTCAAACATC
AY570744.1|  ACATCCCAAAGTT-------------CTGACTTCAAACGTAAAC----------------GACAGAAAT-GCAATTATTATATTGGTTTCACTGATCTTCGTCGTGATTGTATTCATTATAATTTTAGTCGTCAAACATC
AY570742.1|  AGCACCCATTGATACTTTTGCAATGACAGCCTCCGGAAGTAAAGATGAAAGATCAACATGGACAATAATCGGAATTATTACATTGGTTGTACTGATTTTCGTCGTGATTGGAGGCGGTATAACTGTATTCGTCAAACGTT
AY570748.1|  --ATCAAAATG-------------------------------------------------GAAAATAATCGCAATTATTGCATTGGTTGCACTGGCCATCGTCGTGATTGGAGGCTTTATATATATATGCGTCAAATGAA
AY570756.1|  --ATCAAAATG-------------------------------------------------GAAAATAATCGCAATTGTTGCATCGGTTACACTGATCATCGTCTTGATTGCAGGCGCTGTAGCTAAATTCGGCAATTGTT
 Consensus   a.atCccAatG.t.............c.g.ct.c..a.gtaaa.................GAcAatAATcGcAATTaTTgcATtGGTTgcACTGatcaTCGTCgTGATTGgAggCg.TaTAacT.tAttCGtCAAacgt.

             981     990       1000      1010      1020      1030      1040      1050      1060      1070      1080      1090      1100      1110      1120
             |--------+---------+---------+---------+---------+---------+---------+---------+---------+---------+---------+---------+---------+---------|
AY570737.1|  GTTGACGTTCTTTCACATGACATTTTTGTAATTTATTAAATAATTAATCGTTTTTGTAGTTATTGTAACTGTAGTTTTTAGTAA-ATAAATGCTTATTCTGTTCAAACAGCAAAAAAAAAAAAAAAAAA
AY570753.1|  ATTGACGTTCTTTTACATAACTTTGTTT-TAATTTATTAAATAATTAATCGTTTTTGTAATTATTGTAACTGTAGTTTTTAGTAA-ATACATGCTTATTCTGTTCAAA--------AAAAAAAAAAAAAA
AY570744.1|  ATTGACGTTCTTTTACATAACTTTGTTT-TAATTTATTAAATAATTAATCGTTTTTGTAATTATTGTAACTGTAGTTTTTAGTAA-ATACATGCTTATTCTGTTCAAA----------AAAAAAAAAAAA
AY570742.1|  GTTGATGTT-TCCCATATGACATTTTTTAAATTTATTAAATAATTAATAGTTTTTGTAGTTATTGTTACTGTAGTTTTTAGTAA-ATAAATGCTTATTCTGTTCAAAAAGAAAAAAAAAAAAAAAAAAAAAAAAAAA
AY570748.1|  ATTGTTGGTCTTCTACATAACATTTTTTGAATTAATTAAATAATTGATAGTTTTTGTAGTTATTGTAATTGTAGTTTTTAGTAA-ATACATGCTTATTATGCT-------CAAAAAAAAAAAAAAAAAA
AY570756.1|  GTTGATGTTTTCCCATATGACATTTTTTAAATTAATTAAATAATTGATAGTTTTTGTAGTTATTGTAACTGTAGTTTTTAGTAAGATACATGCTTATTATGCTTAAAAAAAAAAAAAAAAAAAAAAAAA
 Consensus   gTTGatGtTcTtccAcATgACaTTtTTTt.AATTtATTAAATAATTaATaGTTTTTGTAgTTATTGTaAcTGTAGTTTTTAGTAA.ATAcATGCTTATTcTGtTcaaa.a..aaaaAAAAAAAAAAAAAa........
```
