# Supplementary figures and images for: Origin of a novel protein-coding gene family with similar signal sequence in Schistosoma japonicum
Source: BMC Genomics. 2012 Jun 20;13:260. doi: 10.1186/1471-2164-13-260 (PMC3434034; doi:10.1186/1471-2164-13-260)

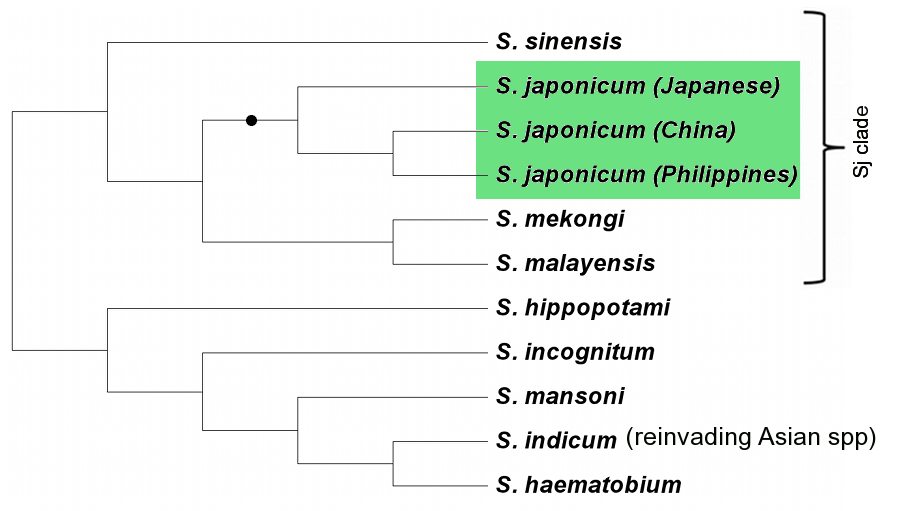

Supplement: Additional file 3 — Phylogenetic tree of the genusSchistosomashowing the possible origination point of new duplicated genes. The species phylogeny was adapted from [53] as inferred from DNA sequencing, comparative molecular genomics and karyotyping. This phylogenetic tree was manually simulated and thus the length of the branches does not estimate dates or time scale. The tree shows the S. japonicum clade and a representative each of the other clades in the genus including the species that reinvaded Asia from Africa. See review in [53]. Based on the result of the southern hybridization in Figure 2, the species and strains that contain the duplicated genes encoding products with similar signal sequence are colored green and we inferred that the most probable time point estimate (black dot) of the gene’s emergence could be after the other species in the S. japonicum group (in parenthesis) have diverged. [file 1471-2164-13-260-S3.tiff]

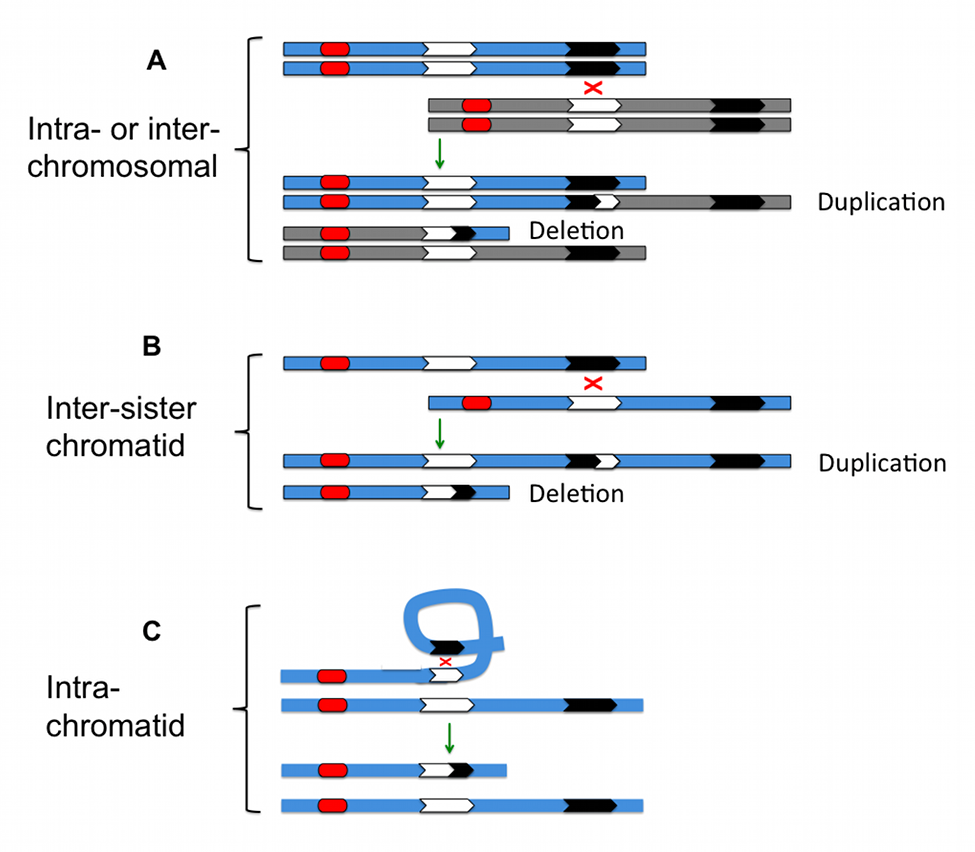

Supplement: Additional file 6 — A simplified illustration of repetitive element mediated DNA level non-allelic homologous recombination (NAHR). Repetitive elements provide the requisite homologous DNA sequence for DNA level recombination between non-allelic pairs by a NAHR mechanism. NAHR can occur within a chromosome (intra-homologous chromosomal), between chromosomes (inter-chromosomal), between sister-chromatids or within a chromatid to give rise to disperse duplicates of the intervening genomic locus. The figure was adapted from [60]. Also see Additional file 1 for a cartoon of NAHR and other mechanisms of new gene origination, and [20,26-29,60] for review. [file 1471-2164-13-260-S6.tiff]

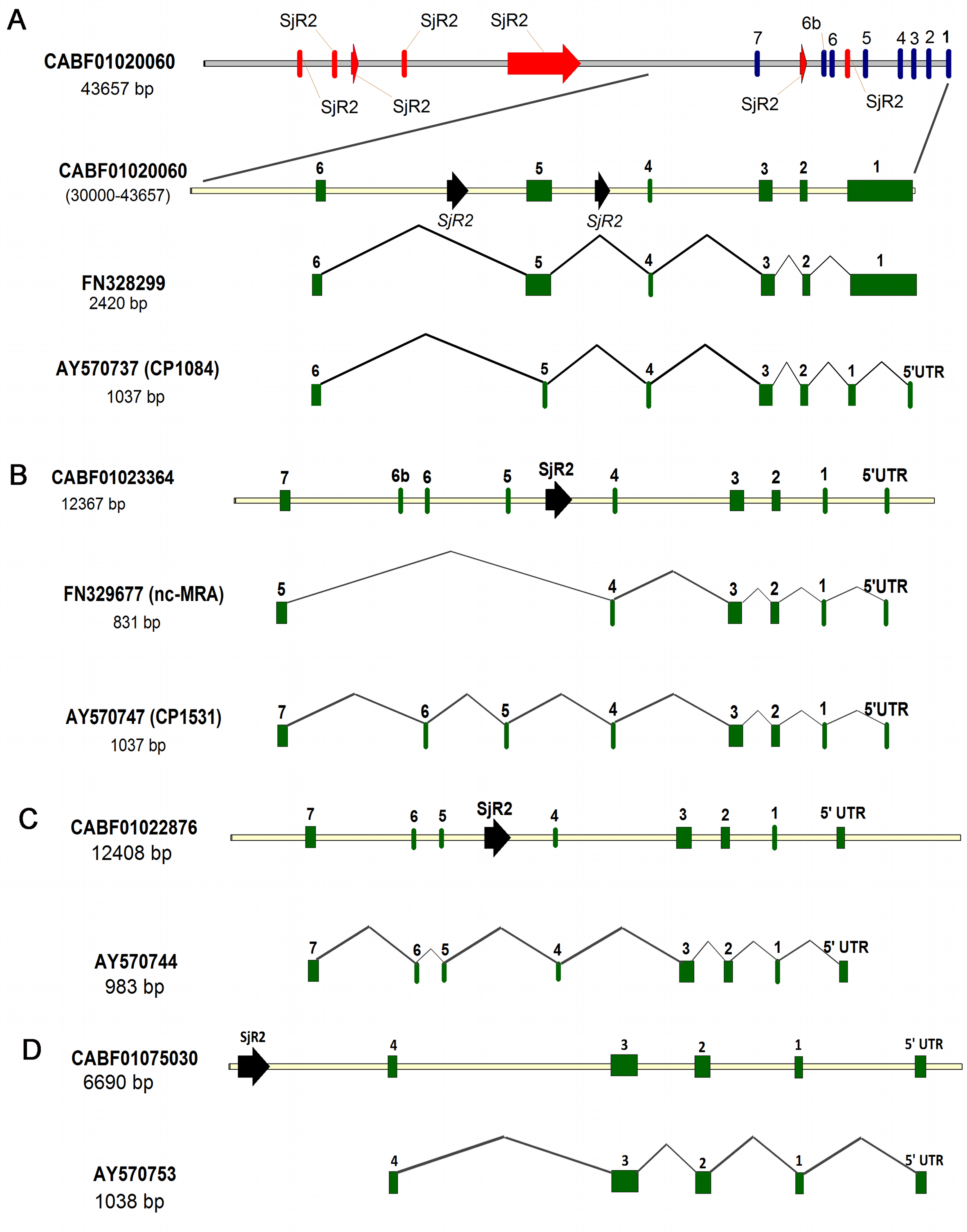

Supplement: Additional file 7 — Splicing models of some protein-coding representatives of the young duplicons. Based on gene prediction from the contigs using GeneQuest and GeneMark and alignment of cDNAs to genome sequences using Spling program, we married the predicted products to the transcriptome database of this parasite and found that some of the duplicons are able to code for distinct gene products. Some of the transcription loci can encode two mRNA transcript variants. The significance of this was further explored in Figure 6. [file 1471-2164-13-260-S7.tiff]
