# Supplementary material for: Origin of a novel protein-coding gene family with similar signal sequence in Schistosoma japonicum
Source: BMC Genomics. 2012 Jun 20;13:260. doi: 10.1186/1471-2164-13-260 (PMC3434034; doi:10.1186/1471-2164-13-260)
Supplement: Additional file 4 — Expected fragments on restriction map of genome scaffolds correspond to bands on southern blots. To confirm dispersed duplication hypothesis and to exclude the possibilty of overlapping among the loci, the restriction map of six of the genome scaffolds bearing duplicated loci were generated (A). Using same restriction endonuclease enzymes as in the generated maps, we performed southern hybridization using restriction digested genomic DNA from S. japonicum species and strains, and were able to match the expected fragment sizes with the observed bands on the hybridization blots. The contigs and the expected probe binding sites were labeled followed by their sequence ranges. We denoted the respective restriction digested fragments with probe binding site using alphabets with their expected restriction digestion product sizes in parenthesis (E + E: EcoRI + EcoRV; E + H: EcoRI + HindIII; B + H = BamHI + HindIII). As shown in (B), we were able to match the expected fragment with the southern blot bands, labeled using their corresponding alphabetic codes. Probe binding site on the positive strand were colored ‘green’ while the antisense sites were colored ‘red’. The tiny vertical lines on the graphics represent the cutting sites of the selected restriction enzymes. The restriction map and the image were generated using DNADynamo sequence analysis software. [file 1471-2164-13-260-S4.ppt]

## Slide 1
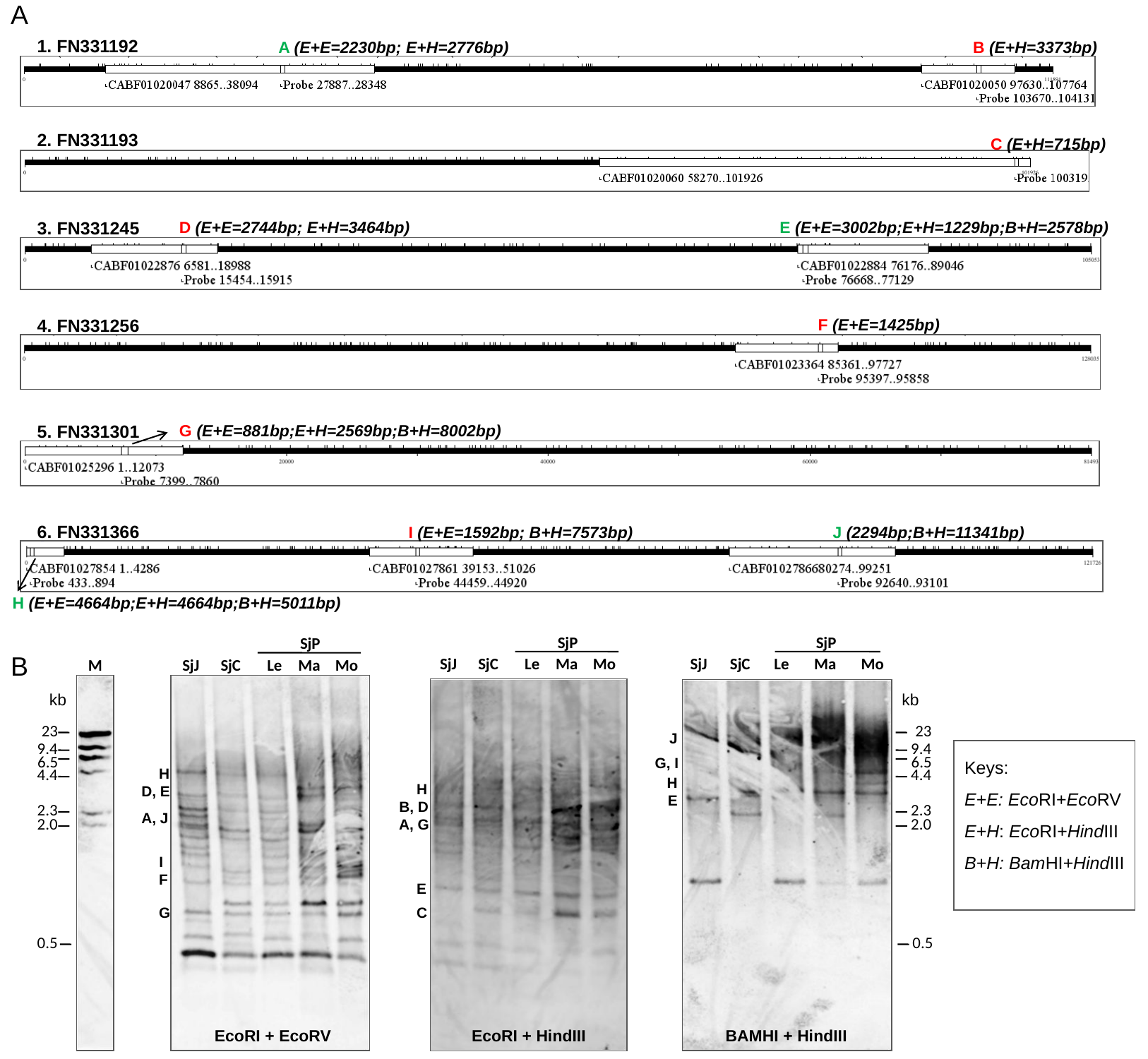

A
1. FN331192
A (E+E=2230bp; E+H=2776bp)
B (E+H=3373bp)
2. FN331193
C (E+H=715bp)
3. FN331245
D (E+E=2744bp; E+H=3464bp)
E (E+E=3002bp;E+H=1229bp;B+H=2578bp)
4. FN331256
F (E+E=1425bp)
G (E+E=881bp;E+H=2569bp;B+H=8002bp)
5. FN331301
6. FN331366
I (E+E=1592bp; B+H=7573bp)
J (2294bp;B+H=11341bp)
H (E+E=4664bp;E+H=4664bp;B+H=5011bp)
SjP
SjP
SjP
B
SjJ
SjC
Le
Ma
Mo
SjJ
SjC
Le
Ma
Mo
M
SjJ
SjC
Le
Ma
Mo
kb
kb
23
23
J
9.4
9.4
Keys:
E+E: EcoRI+EcoRV
E+H: EcoRI+HindIII
B+H: BamHI+HindIII
6.5
6.5
G, I
4.4
H
4.4
H
H
D, E
E
B, D
2.3
2.3
A, J
2.0
2.0
A, G
I
F
E
G
C
0.5
0.5
EcoRI + EcoRV
EcoRI + HindIII
BAMHI + HindIII
