# Supplementary material for: Origin of a novel protein-coding gene family with similar signal sequence in Schistosoma japonicum
Source: BMC Genomics. 2012 Jun 20;13:260. doi: 10.1186/1471-2164-13-260 (PMC3434034; doi:10.1186/1471-2164-13-260)
Supplement: Additional file 8 — SjCP1084 protein coding mRNA [GenBank:AY570737] and a non-coding transcript [GenBank:FN328299] are products of alternative splicing.Based on gene prediction from the contigs using GeneQuest and GeneMark, and alignment of cDNAs to genome sequences using Splingprogram, we observed that two mRNA transcript variants were produced from [GenBank:CABF01020060]. This figure is same as Figure 6 (A) but we have in addition presented the aligned sequence of the two transcripts showing details of alternative splicing. An extra splice site was evolved in the first exon of the non-coding transcript [GenBank:FN328299]. When the splice site is recognized, an ORF encoding SjCP1084 protein coding mRNA [GenBank:AY570737] variant is created. [file 1471-2164-13-260-S8.pdf]

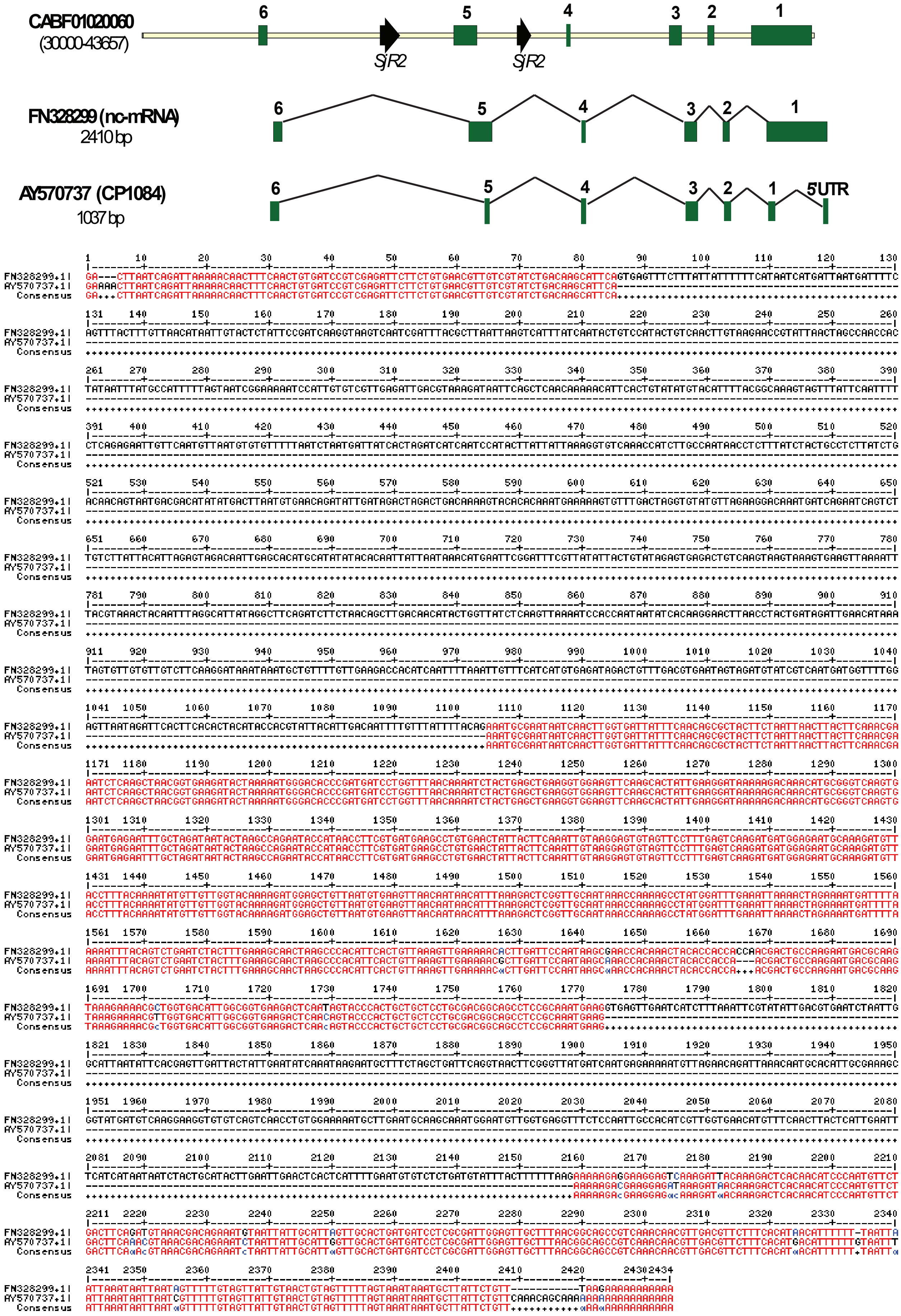
CABF01020060
(30000-43657)
6
5
4
3
2
1
SjR2
SjR2
FN328299 (nc-mRNA)
2410 bp
AY570737 (CP1084)
1037 bp
5'UTR
FN328299.1
AY570737.1
Consensus
