# Supplementary material for: Origin of a novel protein-coding gene family with similar signal sequence in Schistosoma japonicum
Source: BMC Genomics. 2012 Jun 20;13:260. doi: 10.1186/1471-2164-13-260 (PMC3434034; doi:10.1186/1471-2164-13-260)
Supplement: Additional file 9 — SjCP1531 protein coding mRNA [GenBank:AY570742] and a non-coding transcript [GenBank:FN329677] are products of alternative splicing. Based on gene prediction from the contigs using GeneQuest and GeneMark, and alignment of cDNAs to genome sequences using Spling program, we observed that two mRNA transcript variants were produced from [GenBank:CABF01023364]. This figure is same as Figure 6 (B) but we have in addition provided the aligned sequence of the two transcripts showing details of alternative splicing. Two extra splice sites were not utilized in the transcription of the non-coding transcript [GenBank:FN329677]. When the splice sites were recognized, exons 5 and 6 of a translatable ORF were created to produce SjCP1531 protein coding mRNA [GenBank:AY570742] variant. Refer to RT-PCR result in Figure 5 (C) where two bands (exact size and sequence as the two variants described above) are seen on the agarose gel electrophoresis image. [file 1471-2164-13-260-S9.pdf]

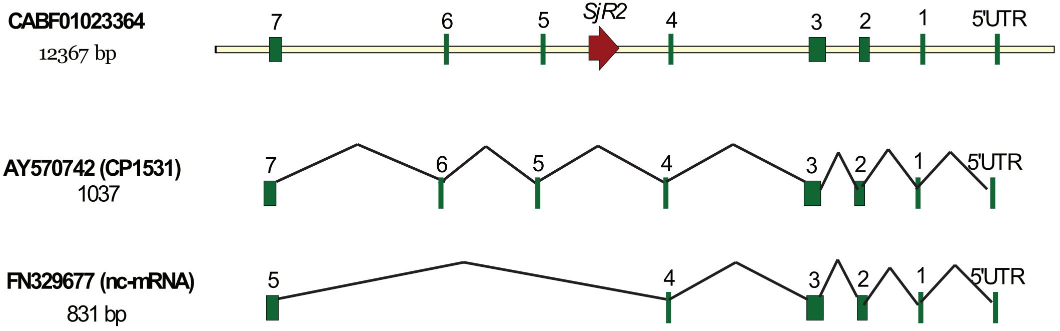


```
           1       10        20        30        40        50        60        70        80        90        100       110       120       130
           |--------+---------+---------+---------+---------+---------+---------+---------+---------+---------+---------+---------+---------|
AY570742.1|GAAAAGCCTAATCAGATTACTATCATCCGTCAGCTGTGATCCGTCGGGATTCTTCTGTGAACCTTTTTGTATCTGTTAAATGTTTAAAGTGCGAATTATCAACTTGGTGAATATTTCAACTGTGCTACTT
FN329677.1|----AGCCTAATCAGATTACTATCATCCGTCAGCTGTCATCCGTCGGGATTCTTCTGTGAACCTTTTTGTATCTGACAAATGTTTAAAATGCGAATTATCAACTTGGTGATTATTTCAACTGTGCTACTT
 Consensus ++++AGCCTAATCAGATTACTATCATCCGTCAGCTGTcATCCGTCGGGATTCTTCTGTGAACCTTTTTGTATCTGacAAATGTTTAAAaTGCGAATTATCAACTTGGTGAaTATTTCAACTGTGCTACTT

           131     140       150       160       170       180       190       200       210       220       230       240       250       260
           |--------+---------+---------+---------+---------+---------+---------+---------+---------+---------+---------+---------+---------|
AY570742.1|CTGATTAACTTACTTCAAACGAAATCTCAAGTTAACGTTGAACAACTTGTAACTGTCCTGGAGGTAAACATCAAAGCCCAATTAAAGAATAAAACGACAGAAGTTTTGGTCGAGTGGAATCCGCTTTACC
FN329677.1|CTGATTAACTTACTTCAAACAAAATCTCAAGGTAACGGTGAACAACATGAAACTGTGCTGGAGGTAAACATCAAAGCACAATTAAAGAATAAAACGACAAAACTTTTGGTCGAGTGGAATCCGCTTCACC
 Consensus CTGATTAACTTACTTCAAACaAAATCTCAAGgTAACGgTGAACAACaTGaAACTGTcCTGGAGGTAAACATCAAAGCaCAATTAAAGAATAAAACGACAaAAcTTTTGGTCGAGTGGAATCCGCTTcACC

           261     270       280       290       300       310       320       330       340       350       360       370       380       390
           |--------+---------+---------+---------+---------+---------+---------+---------+---------+---------+---------+---------+---------|
AY570742.1|AAGACAATTCTATGCCAGAATACCGTAAACTTCTTGATGAAACCTGTAAACTATTTCTTCCATTTGTAAACGAAAAAGTTCCTCCGAGTACATATGGTGGTGAATGCACAAATGCTAACTTTAGTGAAGC
FN329677.1|AAGATAATTCTATGCCAGAAAACCATGAACTTCTTGATGAAGCCTGTAAACTATTTCTTCCAGTTGTAAACAAAAAAGTTCCCCCGAGTAAATATGTTGGTGAATGCACAAATGCTAACTTTAATGAACC
 Consensus AAGAcAATTCTATGCCAGAAaACCaTaAACTTCTTGATGAAaCCTGTAAACTATTTCTTCCAgTTGTAAACaAAAAAGTTCCcCCGAGTAaATATGgTGGTGAATGCACAAATGCTAACTTTAaTGAAcC

           391     400       410       420       430       440       450       460       470       480       490       500       510       520
           |--------+---------+---------+---------+---------+---------+---------+---------+---------+---------+---------+---------+---------|
AY570742.1|TCCACAAGATGGATCATATTCTCGTGTTAATGCGGAGTTACGTTTAATATATAAATACTCCGATAATCCACAAATTCAGACTGACAGGTTGCAGGATGACTTGAACGATCGAGAGGAATTCATCACATCC
FN329677.1|TCCACAAGATGGAACAAATTCTCGTGTTAATGCGGAGTTACGTTTAATATATAAATACTCCGCTGATCCACAAGTTCAAACTGACAAGTTGCAGAATGACTTGAACGAGCAAGAAGAATTCATCAGCTCT
 Consensus TCCACAAGATGGAaCAaATTCTCGTGTTAATGCGGAGTTACGTTTAATATATAAATACTCCGaTaATCCACAAaTTCAaACTGACAaGTTGCAGaATGACTTGAACGAgCaAGAaGAATTCATCAcaTCc

           521     530       540       550       560       570       580       590       600       610       620       630       640       650
           |--------+---------+---------+---------+---------+---------+---------+---------+---------+---------+---------+---------+---------|
AY570742.1|GAGTCTTCTTTCGTAGCATATAATCCCAAATTCACTGTTAAAATAGAAAAACTCTTCATTCCAATAAGCGAATCACAAACTACACCACCACCAACGACTGCCAAGAATGACGCAAGTAAAAAAGACGATG
FN329677.1|GAGTCTTCTTTGGTAGCATATAATCCCGAATTCACAGTTGAAGTACGAACATACGCGATTCCAATAAGCGAACAGCAAACTACACCACCATCGATGACCGCCAAGAATGACACAA---------------
 Consensus GAGTCTTCTTTcGTAGCATATAATCCCaAATTCACaGTTaAAaTAcaAAaAcaCgccATTCCAATAAGCGAAcaaCAAACTACACCACCAcCaAcGACcGCCAAGAATGACaCAA+++++++++++++++

           651     660       670       680       690       700       710       720       730       740       750       760       770       780
           |--------+---------+---------+---------+---------+---------+---------+---------+---------+---------+---------+---------+---------|
AY570742.1|ATGACAATGGCGGTGAAGACTCAAAAGCACCCATTGATACTTTTGCAATGACAGCCTCCGGAAATAAAGGTAAAAAAGACGATGATGACAATGGCGGTGAAGACTCAAAAGCACCCATTGATACTTTTGC
FN329677.1|----------------------------------------------------------------------------------------------------------------------------------
 Consensus ++++++++++++++++++++++++++++++++++++++++++++++++++++++++++++++++++++++++++++++++++++++++++++++++++++++++++++++++++++++++++++++++++

           781     790       800       810       820       830       840       850       860       870       880       890       900       910
           |--------+---------+---------+---------+---------+---------+---------+---------+---------+---------+---------+---------+---------|
AY570742.1|AATGACAGCCTCCGGAAGTAAAGATGAAAGATCAACATGGACAATAATCGGAATTATTACATTGGTTGTACTGATTTTCGTCGTGATTGGAGGCGGTATAACTGTATTCGTCAAACGTTGTTGATGTTTC
FN329677.1|-----------------------ATGAAGGATCAACATGGACAAAAAGCGGAATTATTGCATTGGTTGCACTGATCATCGTCGTGATTGTAGTCGCTTTAATTGCAGTCGTCAAATGTTGTTGATGTTTT
 Consensus +++++++++++++++++++++++ATGAAaGATCAACATGGACAAaAAgCGGAATTATTaCATTGGTTGcACTGATcaTCGTCGTGATTGgAGgCGcTaTAAcTGcAgTCGTCAAAcGTTGTTGATGTTTc

           911     920       930       940       950       960       970       980       990       1000      1010      1020      1030     1038
           |--------+---------+---------+---------+---------+---------+---------+---------+---------+---------+---------+---------+--------|
AY570742.1|-CCATATGACATTTTTTTAAATTTATTAAAATAATTAATAGTTTTTGTAGTTATTGTTACTGTAGTTTTTAGTAAATAAATGCTTATTCTGTTCAAAAAGAAAAAAAAAAAAAAAAAAAAAAAAAAAAA
FN329677.1|TCCACATAACATTTGTTTTAATTTATTGAATAATTAATCGTTTTTGTAGTTATTGTAACTGTAGTTTTTAGTAAATACATGCTTATTCTGTT-----------------------------------G
 Consensus +CCAcATaACATTTgTTTaAATTTATTaAATAATTAATaGTTTTTGTAGTTATTGTaACTGTAGTTTTTAGTAAATAaATGCTTATTCTGTT+++++++++++++++++++++++++++++++++++a
```
