# Supplementary material for: Origin of a novel protein-coding gene family with similar signal sequence in Schistosoma japonicum
Source: BMC Genomics. 2012 Jun 20;13:260. doi: 10.1186/1471-2164-13-260 (PMC3434034; doi:10.1186/1471-2164-13-260)
Supplement: Additional file 10 — Simulations using our raw data to show DNA-Level recombination mediated by REs by NAHR mechanism.This Powerpoint presentation was used to create the movie in (Additional file 5). [file 1471-2164-13-260-S10.ppt]

## Slide 1
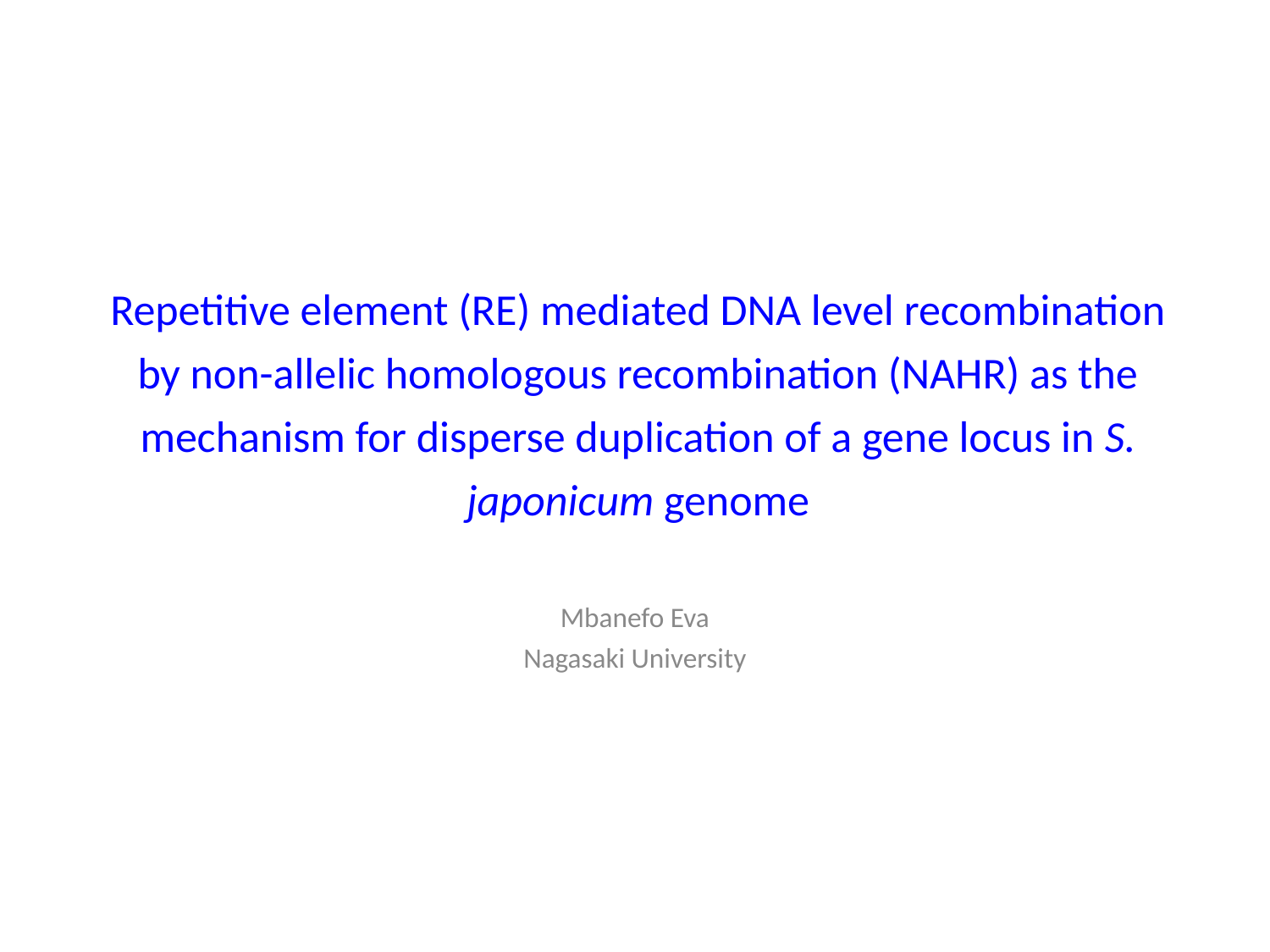

# Repetitive element (RE) mediated DNA level recombination by non-allelic homologous recombination (NAHR) as the mechanism for disperse duplication of a gene locus in S. japonicum genome
Mbanefo Eva
Nagasaki University

## Slide 2
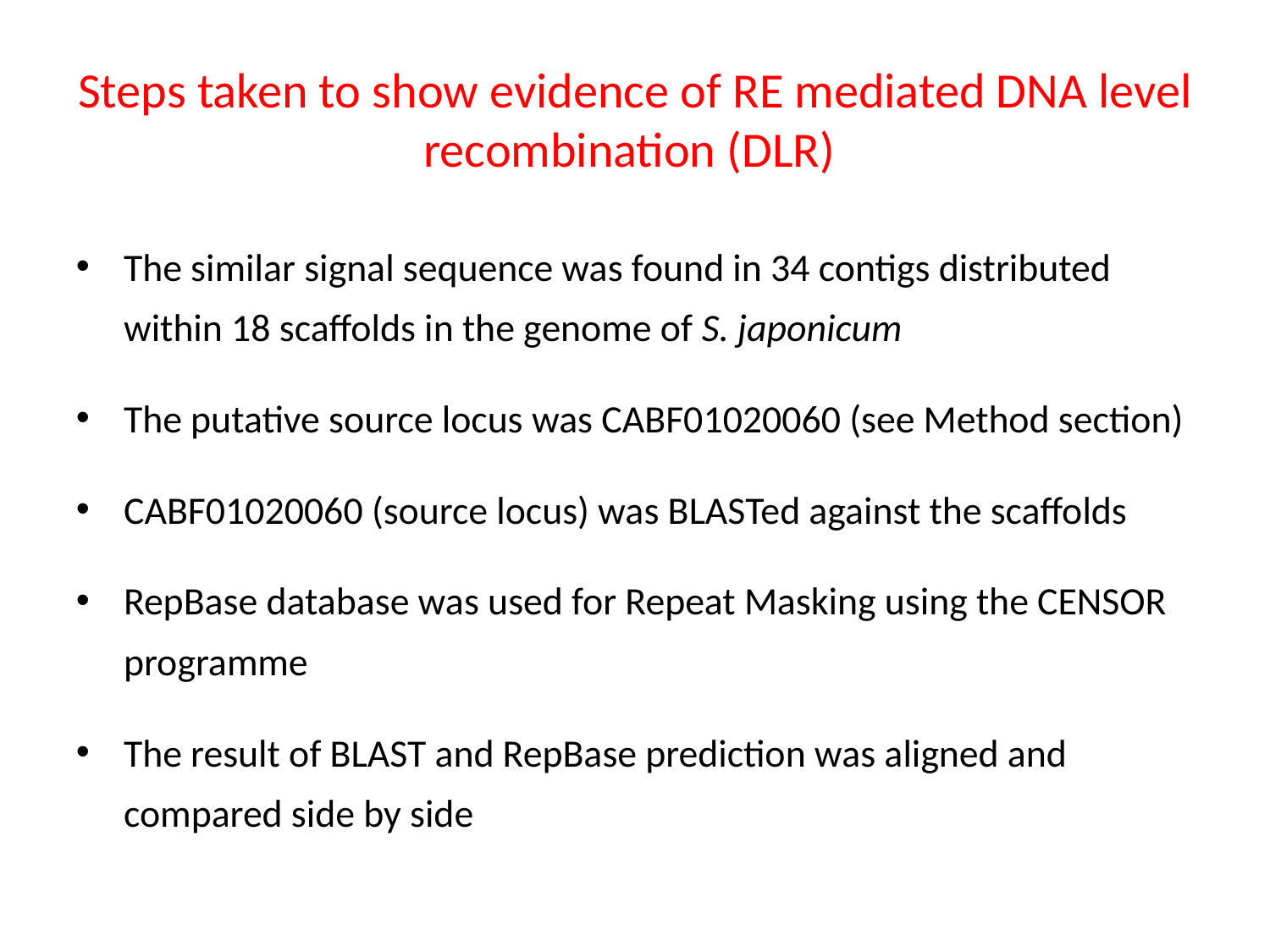

# Steps taken to show evidence of RE mediated DNA level recombination (DLR)
The similar signal sequence was found in 34 contigs distributed within 18 scaffolds in the genome of S. japonicum
The putative source locus was CABF01020060 (see Method section)
CABF01020060 (source locus) was BLASTed against the scaffolds
RepBase database was used for Repeat Masking using the CENSOR programme
The result of BLAST and RepBase prediction was aligned and compared side by side

## Slide 3
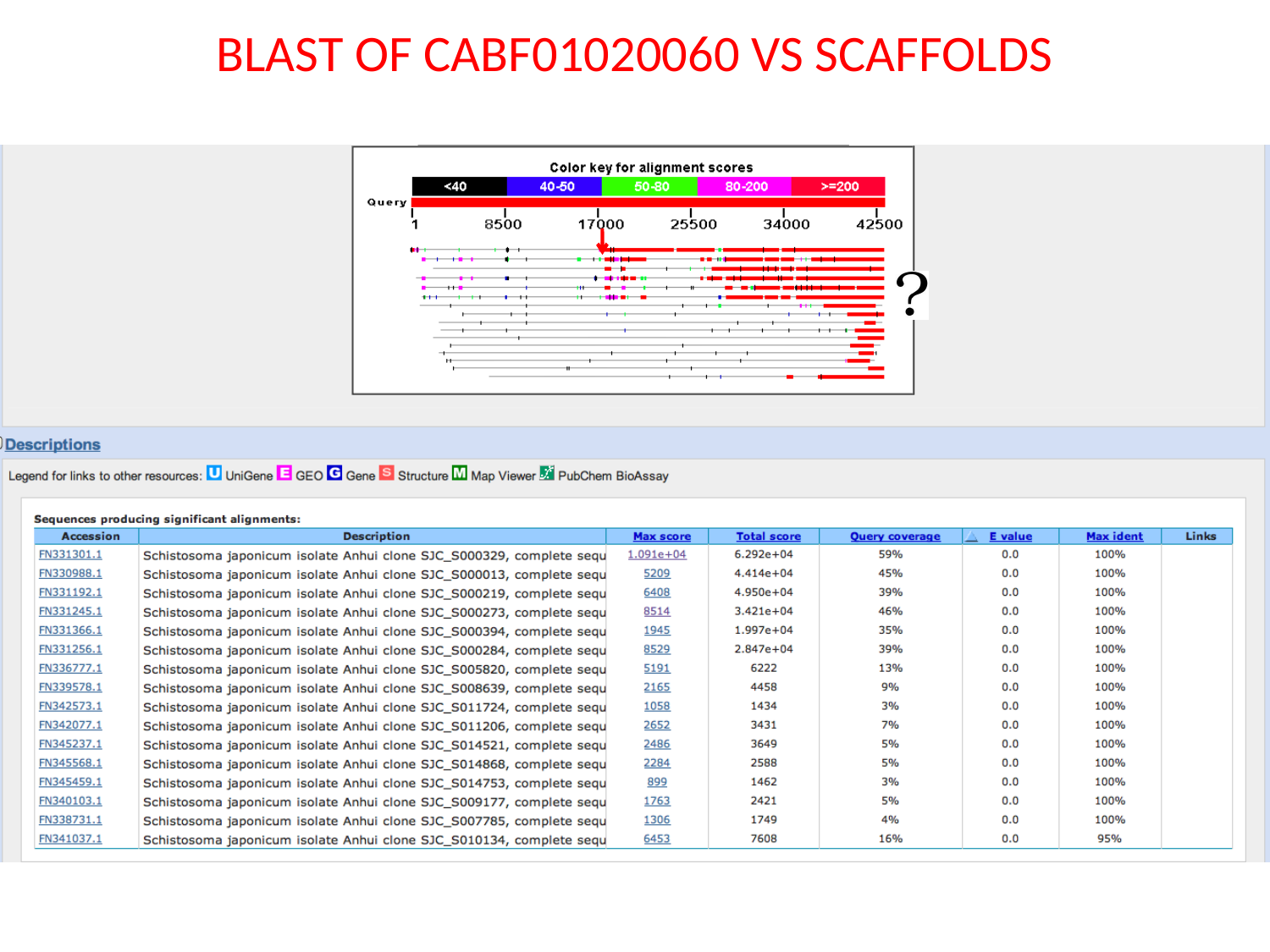

# BLAST OF CABF01020060 VS SCAFFOLDS

## Slide 4
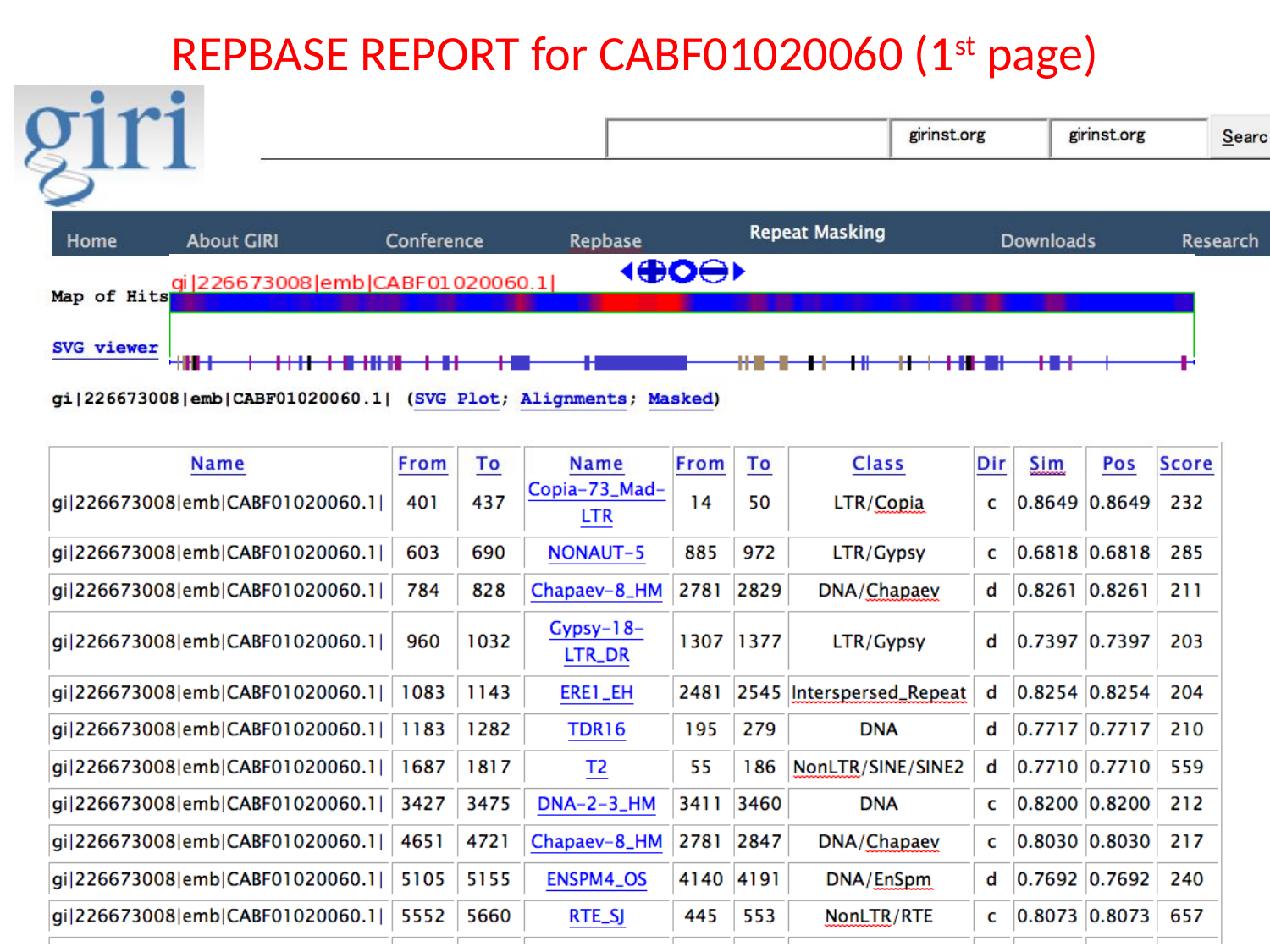

# REPBASE REPORT for CABF01020060 (1st page)

## Slide 5
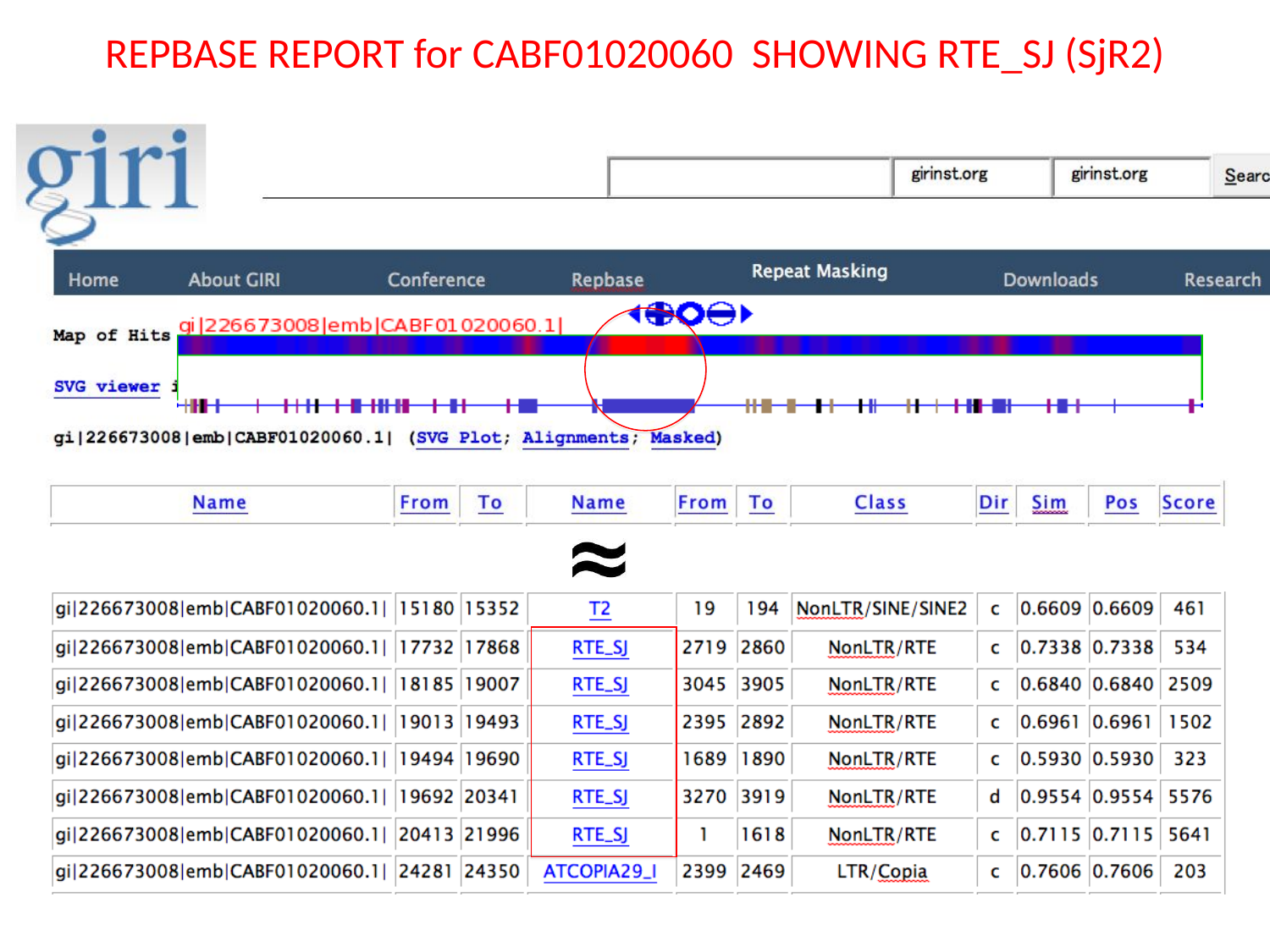

# REPBASE REPORT for CABF01020060 SHOWING RTE_SJ (SjR2)

## Slide 6
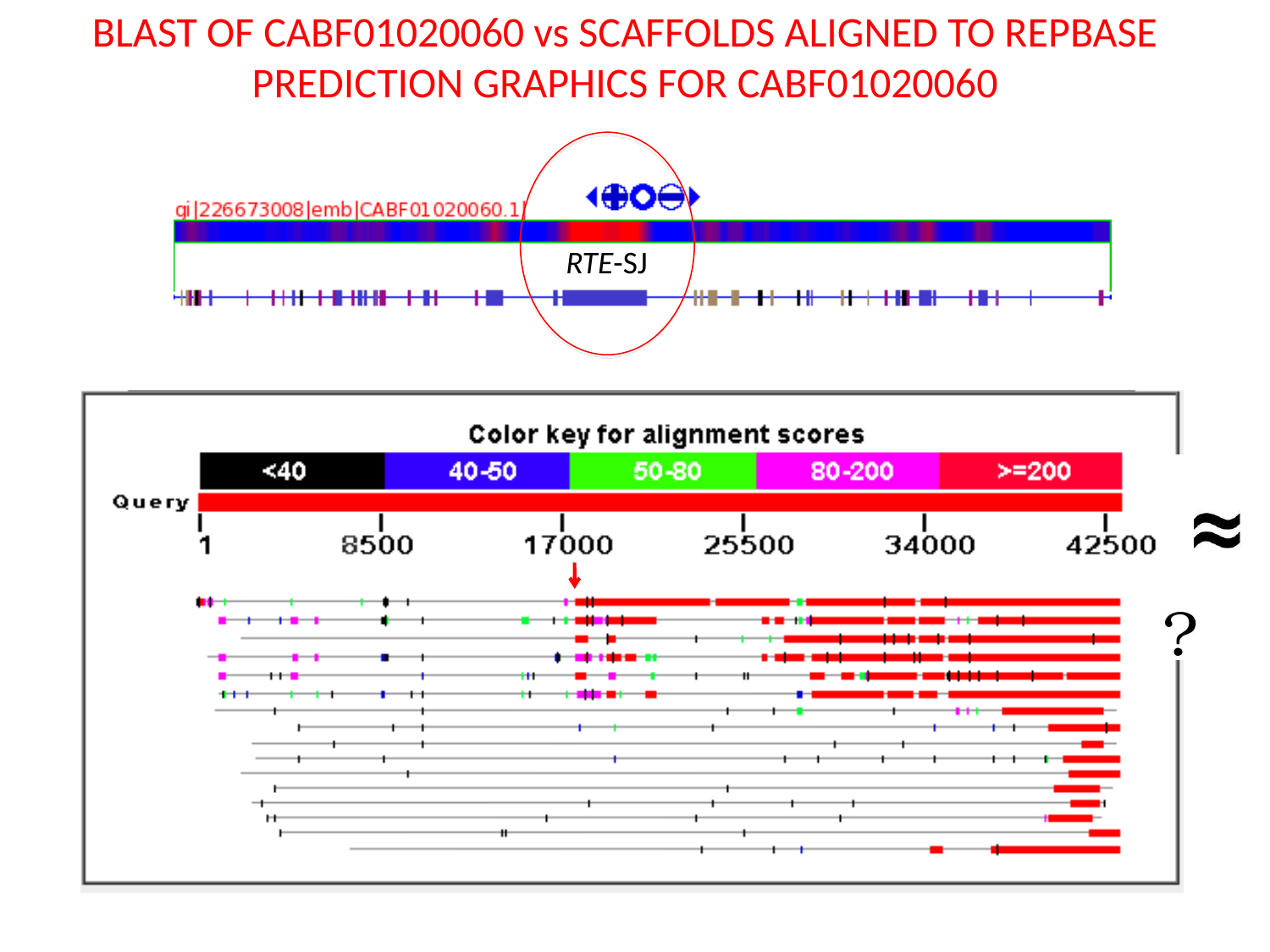

BLAST OF CABF01020060 vs SCAFFOLDS ALIGNED TO REPBASE PREDICTION GRAPHICS FOR CABF01020060
RTE-SJ

## Slide 7
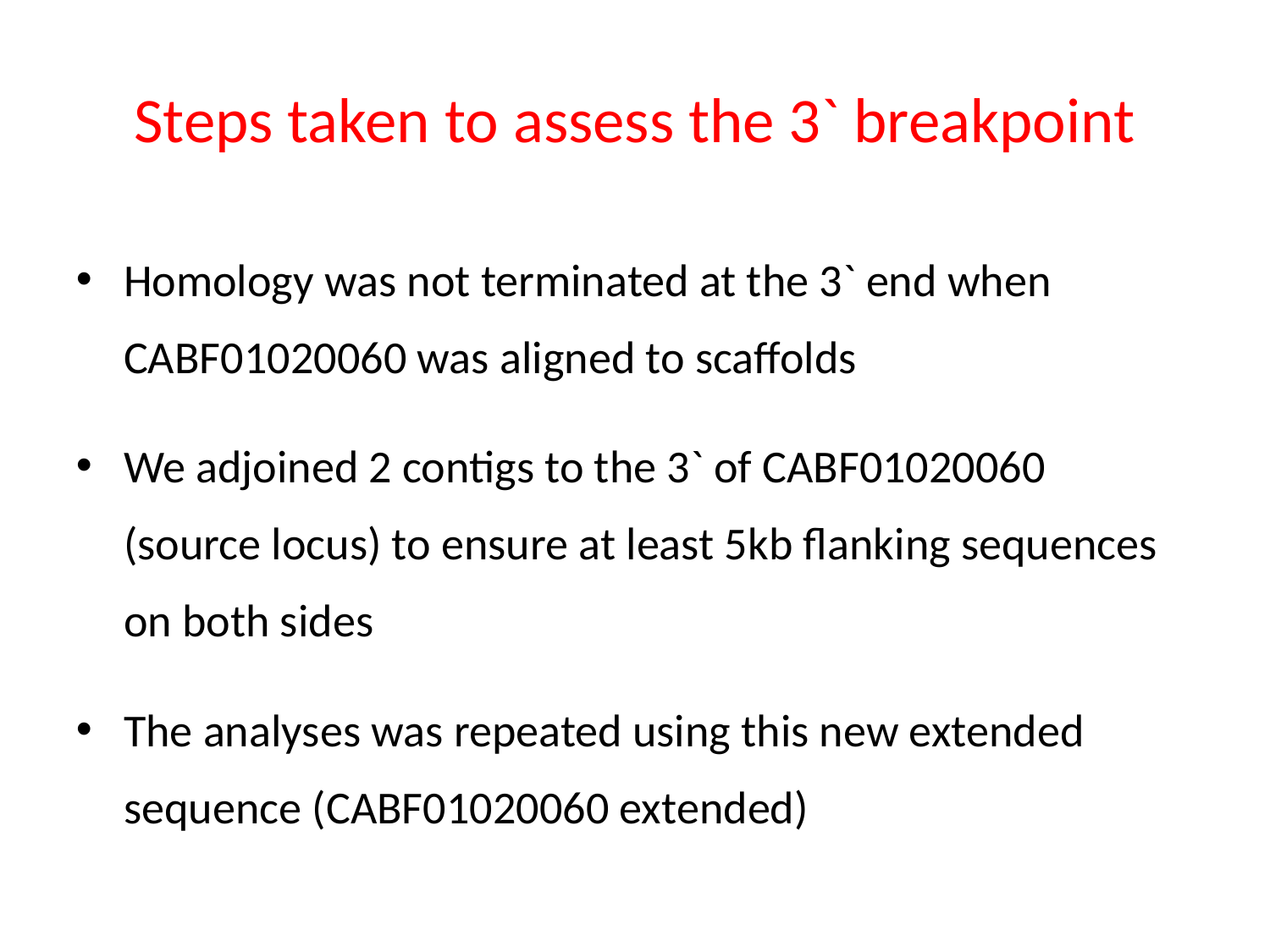

# Steps taken to assess the 3` breakpoint
Homology was not terminated at the 3` end when CABF01020060 was aligned to scaffolds
We adjoined 2 contigs to the 3` of CABF01020060 (source locus) to ensure at least 5kb flanking sequences on both sides
The analyses was repeated using this new extended sequence (CABF01020060 extended)

## Slide 8
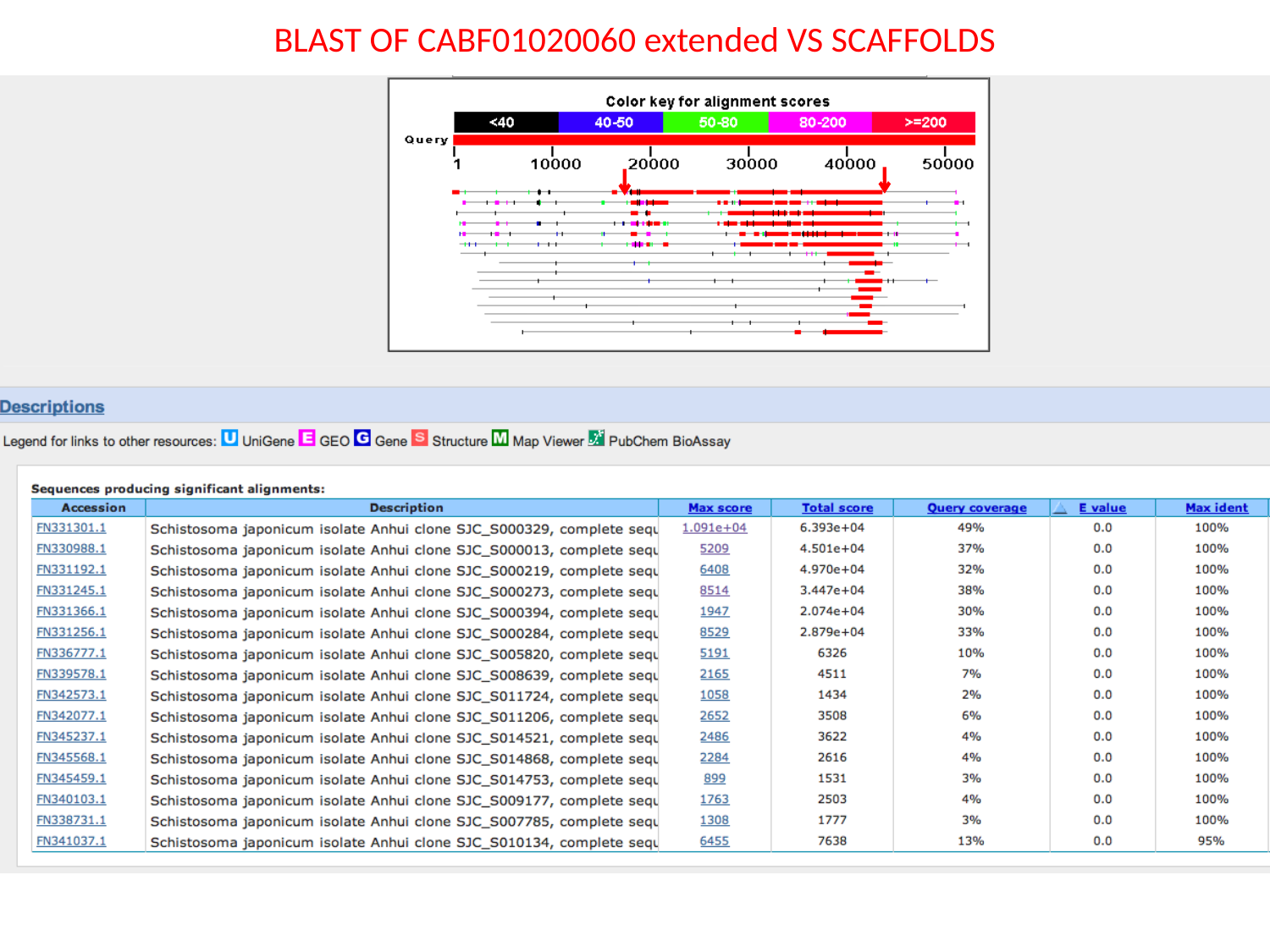

# BLAST OF CABF01020060 extended VS SCAFFOLDS

## Slide 9
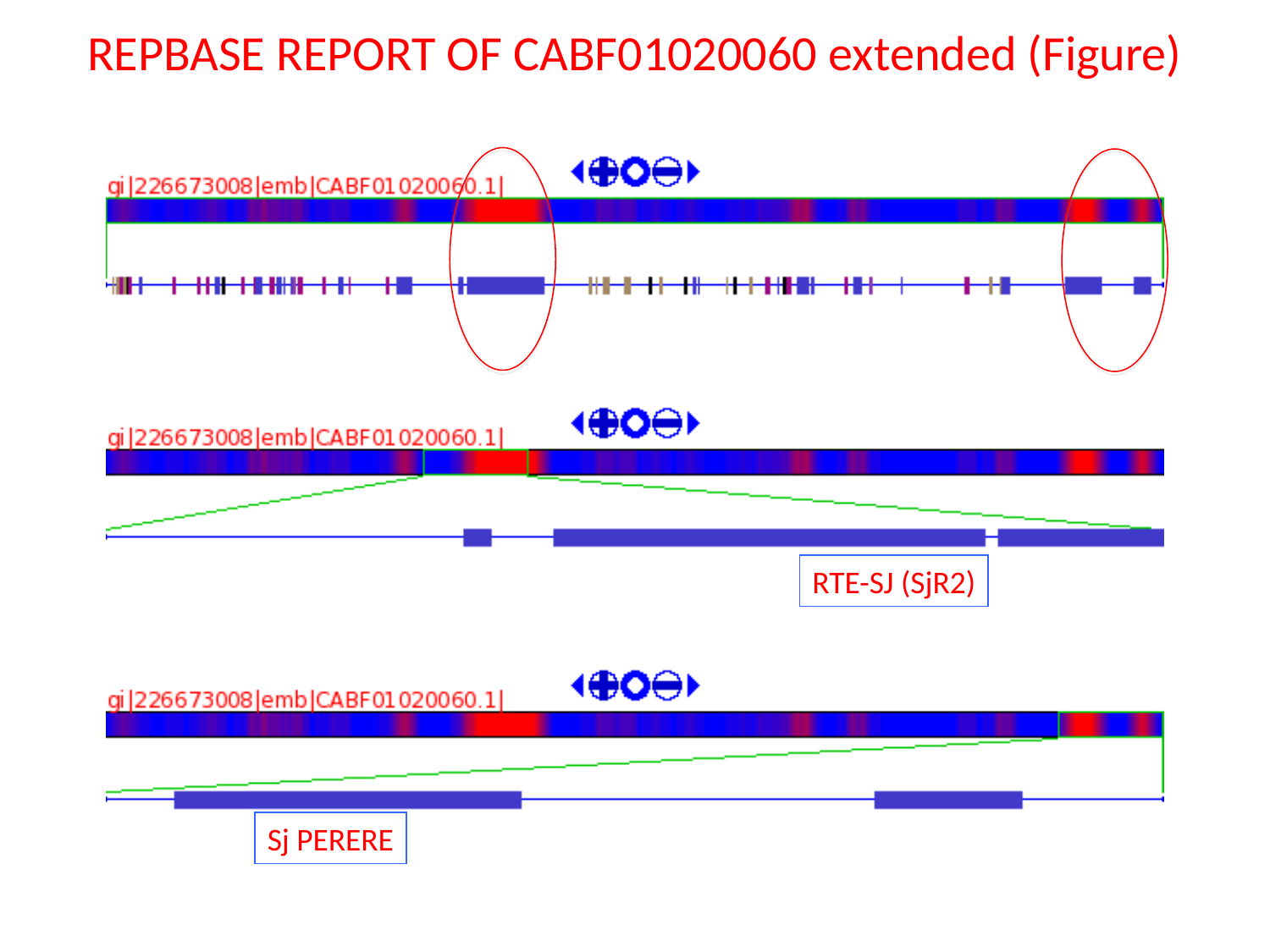

# REPBASE REPORT OF CABF01020060 extended (Figure)
RTE-SJ (SjR2)
Sj PERERE

## Slide 10
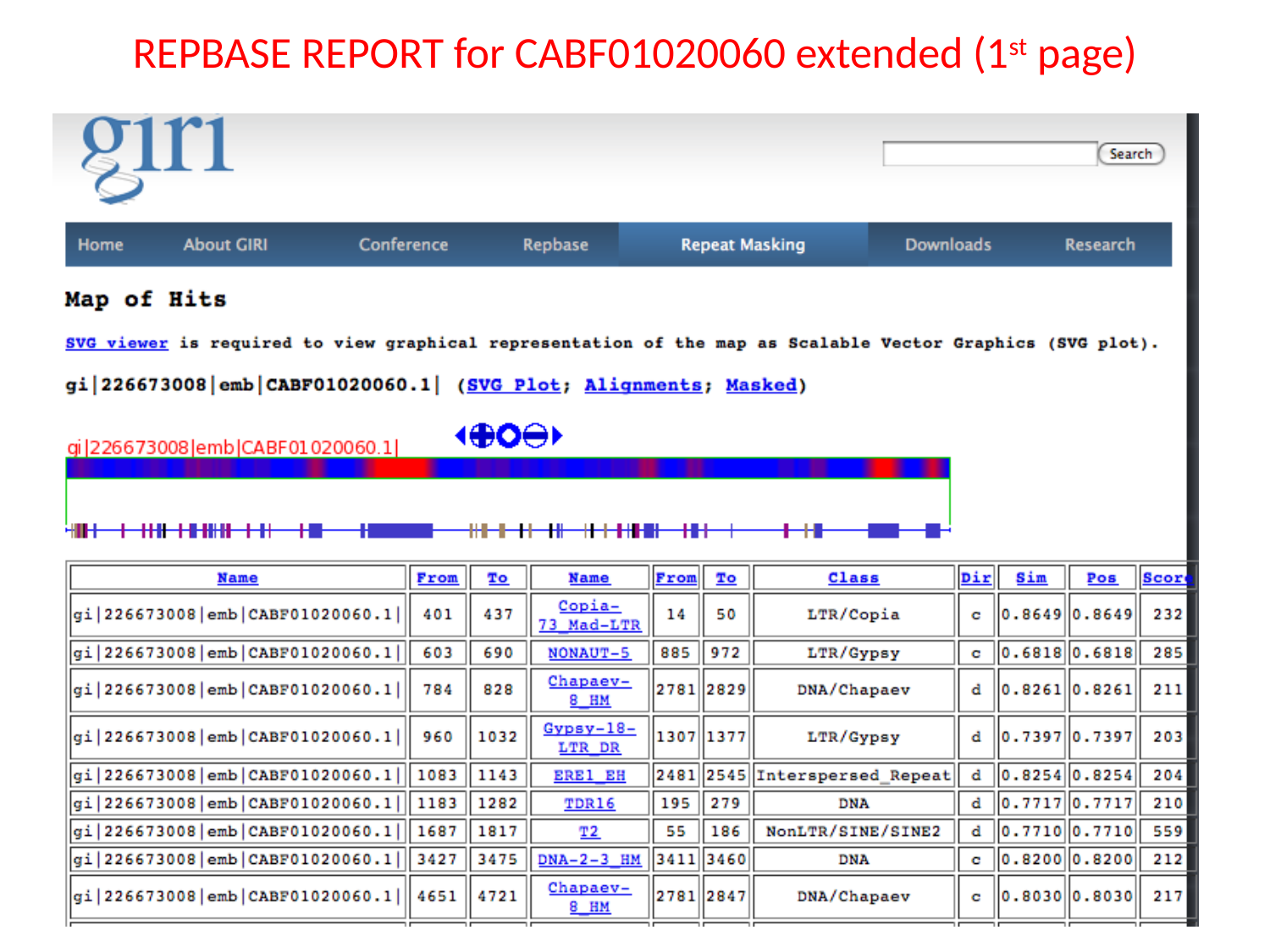

# REPBASE REPORT for CABF01020060 extended (1st page)

## Slide 11
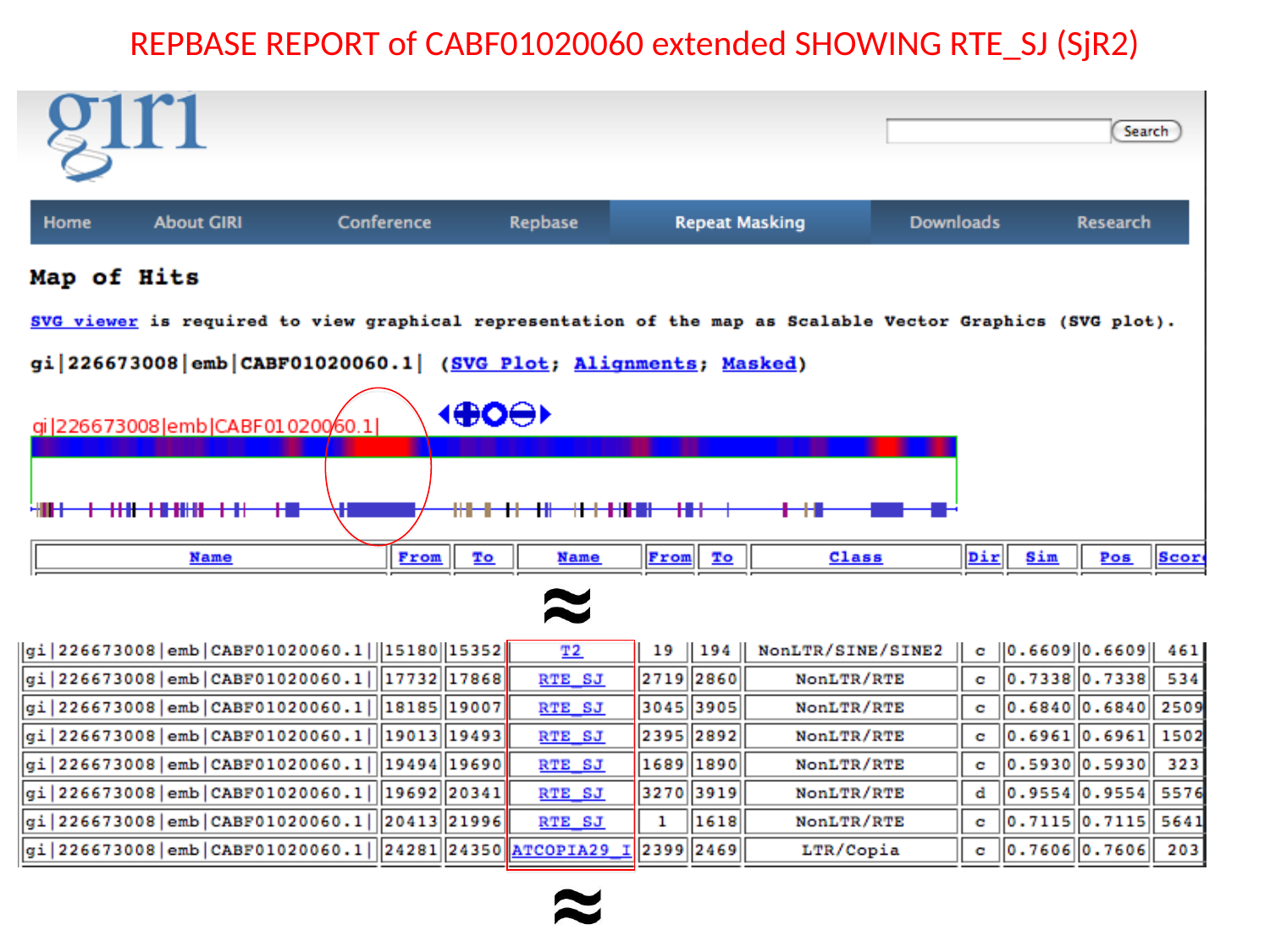

# REPBASE REPORT of CABF01020060 extended SHOWING RTE_SJ (SjR2)

## Slide 12
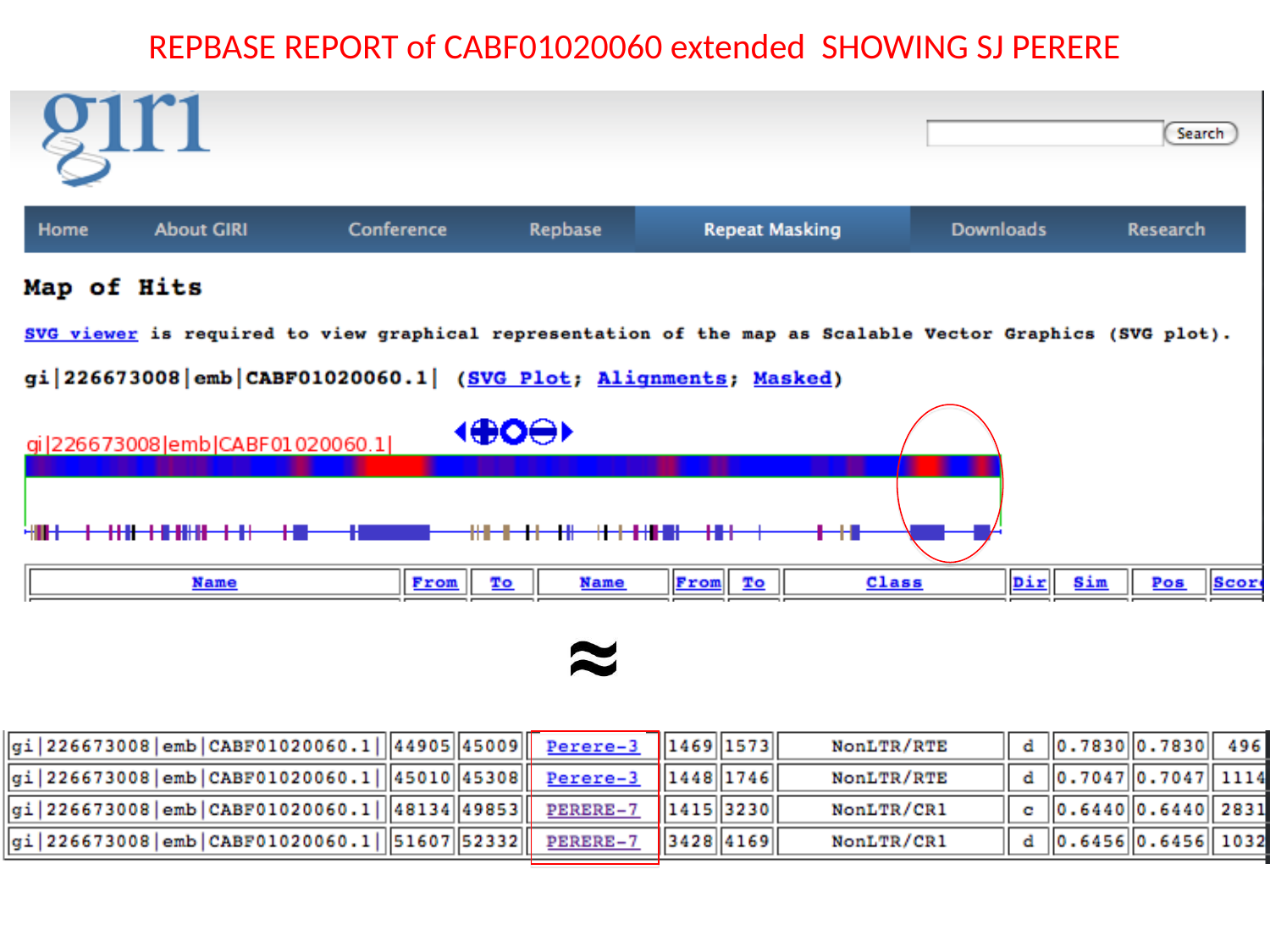

# REPBASE REPORT of CABF01020060 extended SHOWING SJ PERERE

## Slide 13
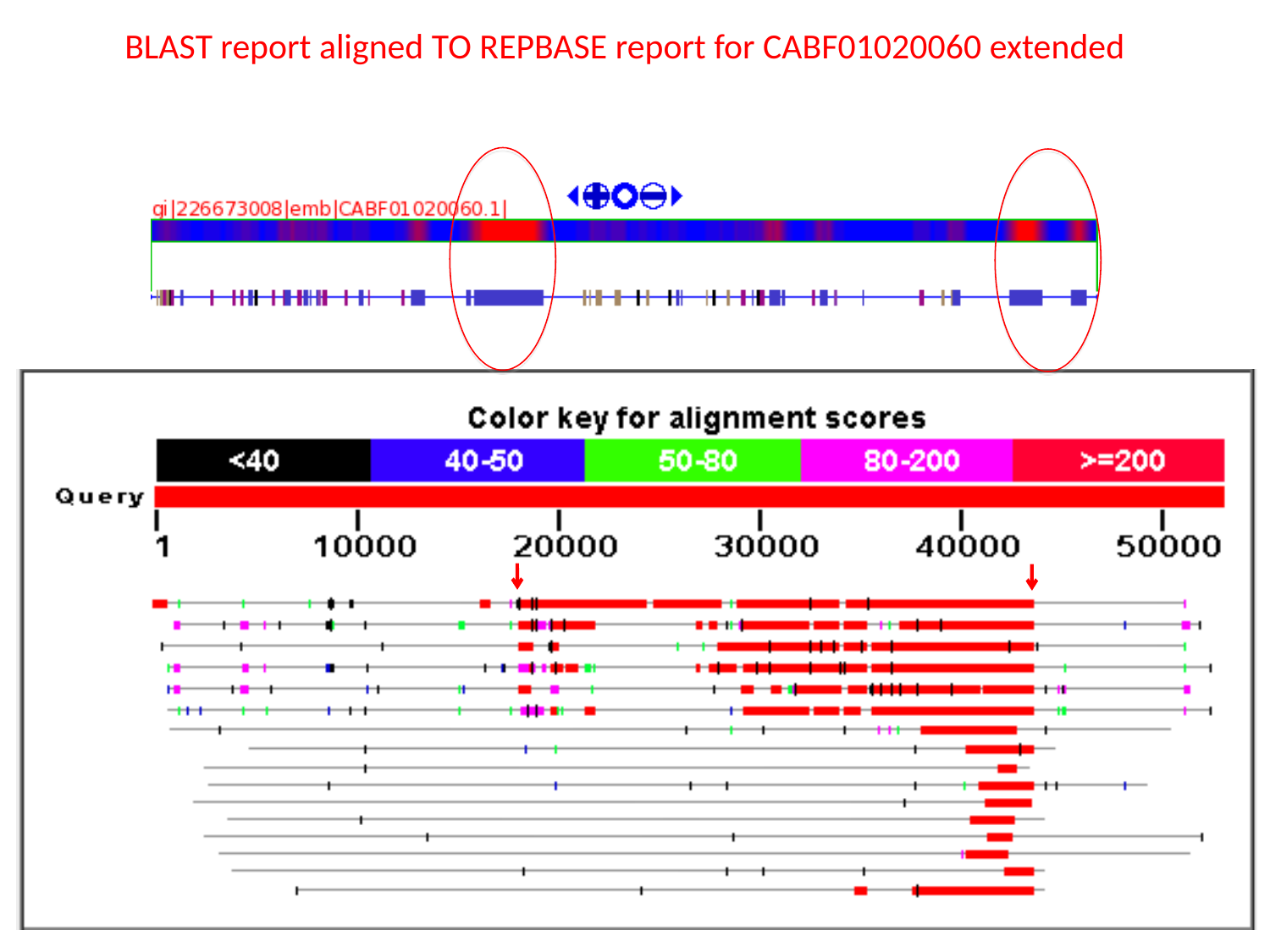

BLAST report aligned TO REPBASE report for CABF01020060 extended
